# Dietary modulation of lung lipids influences inflammatory responses to inhaled ozone

**DOI:** 10.1016/j.jlr.2024.100630

**Published:** 2024-08-30

**Authors:** Russell Hunter, Brenna Baird, Milad Mazloumi-Bakhshayesh, Siem Goitom, Selita Lucas, Guy Herbert, David Scieszka, Edward Davis, Haiwei Gu, Yan Jin, Barry E. Bleske, Matthew J. Campen

**Affiliations:** 1 Department of Pharmaceutical Sciences, University of New Mexico College of Pharmacy, Albuquerque, New Mexico, USA; 2 University of New Mexico Prevention Research Center, University of New Mexico, Albuquerque, New Mexico, USA; 3 Center for Translational Sciences, Florida International University, Port St. Lucie, Florida, USA; 4 Department of Pharmacy Practice and Administrative Sciences, University of New Mexico, Albuquerque, New Mexico, USA

**Keywords:** toxicology, triglycerides, phospholipids, oxidized lipids, omega-3 fatty acids, dietary fat, ozone, lung

## Abstract

The pulmonary system represents a unique lipidomic environment as it contains cellular membrane-bound lipid species and a specialized reservoir of lipids in the airway epithelial lining fluid. As a major initial point of defense, airway lipids react to inhaled contaminants such as volatile organic compounds, oxides of nitrogen, or ozone (O_3_), creating lipokine signaling that is crucial for both the initiation and resolution of inflammation within the lung. Dietary modulation of eicosanoids has gained increased attention in recent years for improvements to cardiovascular health. The current study sought to examine how dietary supplementation with eicosanoid precursors (*i.e*, oils rich in saturated or polyunsaturated fatty acids) might alter the lung lipid composition and subsequently modify the inflammatory response to ozone inhalation. Our study demonstrated that mice fed a diet high in saturated fatty acids resulted in diet-specific changes to lung lipid profiles and increased cellular recruitment to the lung following ozone inhalation. Bioinformatic analysis revealed an ozone-dependent upregulation of several lipid species, including phosphoserine 37:5. Pathway analysis of lipid species revealed the process of lateral diffusion of lipids within membranes to be significantly altered due to ozone exposure. These results show promising data for influencing pulmonary lipidomic profiles via diet, which may provide a pragmatic therapeutic approach to protect against lung inflammation and damage following pulmonary insult.

Air pollution is a known driver of cardiopulmonary diseases (1) and has been shown epidemiologically to be associated with local and systemic inflammatory conditions, including lung, cardiovascular, and metabolic diseases, increased risks of gestational hypertension, and neuroinflammation (2, 3). Two major types of air pollution, ozone (O_3_) and wildfire smoke, may be inextricably linked to climate change and are predicted to increase in the near future (3, 4, 5, 6, 7). Recent studies have begun examining how dietary supplementation with saturated or polyunsaturated fatty acids may alter acute and systemic responses to the inhalation of O_3_, as an actionable public health recommendation to offset health impacts of these emerging exposures. These studies utilized diets supplemented with olive oil, fish oil, and coconut oil to demonstrate diet specific metabolic changes, as well as vasoprotective effects of fish oil following O_3_ exposure (8, 9). Because the health effects of environmental stressors like air pollution are not specifically “druggable” as with distinct diseases, identifying dietary solutions to protect exposed populations has broad appeal for public health. However, understanding the mechanisms of action of dietary factors—like polyunsaturated fats—is valuable to predicting the protective benefits and select subpopulations who may stand to benefit most.

The lipid fraction of the epithelial lining fluid (ELF) contains a diverse range of lipid species with various biological implications in the lumen of the alveolar units of the lung, where physiochemical as well as cellular interactions may work to modify the composition of this microenvironment. Recent characterization of human ELF lipids revealed glycerophospholipids to be the most abundant lipid type, with glycerophoshochololines (PCs) identified as a major lipid class, accounting for roughly 71% of total ELF lipids. The next highest classes are glycerophosphoethanolamines (PEs) (9.8%) and glycerophosphoglycerols (PGs) (8%). The ELF also contains other molecular classes of lipids, including sphingolipids, sterol lipids, and glycerolipids (10). Besides the airway lining, analysis of human bronchial tissue found the major constituent of total lipid signal to be PCs (36%), with TAGs (14%), Cer (12%), and PE (8%) also having major contributions to overall lipid signal. When compared to murine tracheal epithelial samples, the relative proportion of lipid signal percentage was similar for PC, PI, PS, PE, and PE-O classes; however, the human samples had significantly higher proportions of Cer and PG class lipids, and murine samples had significantly higher proportions of SM and PC-O classes (11). How dietary pulmonary lipid composition is impacted by diet, as well as how these changes influence inflammatory response to environmental stressors and oxidative stress remain poorly understood. Beyond the air-liquid interface, the supporting lung interstitum is home to a wide array of vascular, structural, and immune cells whose cellular membranes as well as inta and pericellular lipids contribute to the greater pulmonary lipidome. Analysis of lung lipids for pulmonary diseases such as lung fibrosis (12, 13), chronic obstructive pulmonary disease (14, 15, 16), and lung cancer (17). The interplay between the lipids with the airspace and lung interstitum, as well as how diet modifies these regions of the lung remains loosely characterized.

Studies on dietary supplementation with oils high in omega-3 polyunsaturated fatty acids (PUFAs) such as fish oils have increased in prevalence following the 2002 American Heart Association statement that fish oil supplements significantly reduced fatal cardiac events (18, 19). More recent revisions to clinical recommendations advise omega-3 PUFA supplementation for secondary prevention of chronic heart disease (CHD) and sudden cardiac death among patients with prevalent CHD, as well as secondary prevention of outcomes in patients with heart failure (20). Beyond cardiac health, the role of PUFA supplementation is also being investigated for potential benefits for respiratory disease such as chronic obstructive pulmonary disease (21) and lung cancer (22). The capability of dietary PUFA supplementation to potentially alter oxidative and/or inflammatory reactions within the context of the lung has implications for many areas on inhalation toxicology.

Utilizing similar dietary and exposure paradigms as those previously reported (8, 9), the current research seeks to complement these studies by examining the role of dietary fatty acids in modulating baseline lung lipidomic and metabolomic profiles, in addition to ascertaining the O_3_-induced molecular alterations. Because O_3_ principally interacts initially with ELF phospholipids (23, 24), we hypothesize that O_3_-induced inflammatory responses are modified due to the initial lipidomic profile conditions and that modulating lipidomic profiling with diet will provide critical insight into the pathological molecular interactions of the lung microenvironment.

## Materials and Methods

### Animals

Female C57BL/6J (Stock No:000664) mice aged 6–8 weeks were ordered from Jackson Labororatory. In total, 8 groups of mice with n = 6 mice per diet per exposure were used. Animals were housed in an Association for Assessment and Accreditation of Laboratory Animal Care (AAALAC)-approved facility at the University of New Mexico Health Sciences Center. Animals were maintained at constant temperature (20–24°C), relative humidity (30%–60%), and on a 12-h light/dark cycle throughout the study. Animals were provided with standard or supplemented mouse chow (described below) and water ad libitum. All animal handling and procedures were conducted humanely and approved by the Institutional Animal Care and Use Committee (IACUC) at the University of New Mexico.

### Fatty acid-enriched diets

Animals were fed with one of four diets from acclimation throughout the duration of the study. The grain-based diet (Teklad Global Soy Protein-Free Extruded Rodent Diet, 2020X) served as the control standard chow diet for this study. This diet excludes soybean meal and alfalpha, making it a reliable diet for optical imaging and limiting phytoestrogens. The three remaining diets all contained a small amount of soy, with the addition of oils chosen for different fatty acid compositions. Diets containing soybean oil (Teklad Custom Diet TD.97184), flaxseed oil (Teklad Custom Diet TD.210291), or coconut oil (Teklad Custom Diet TD.210292) were given for 3 weeks prior to exposures to allow animals to adapt to any microbiota changes. The supplemented diets were exchanged every other day to reduce oxidation, as previously described (8). Dietary information can be found in Table 1. Diets will be abbreviated as follows for the duration of this paper: Standard Chow (SC), Soybean Oil (SO), Flaxseed Oil (FO), and Coconut Oil (CO).

**Table 1:** Comparison of diets in terms of overall nutritional contributors as well as fatty acid percentages

	Standard Chow	Soybean Oil	Flaxseed Oil	Coconut Oil
Protein (%)	19.1	17.7	17.7	17.7
Carbohydrates (%)	47	60.1	60.1	60.1
Fat (%)	6.5	7.2	7.2	7.2
Kcal/g	3.1	3.8	3.8	3.8
6:0 Caproic				0.03
8:0 Caprylic				0.48
10:0 Capric				0.36
12:0 Lauric				2.82
14:0 Myristic				1.08
16:0 Palmitic	0.6	0.88	0.41	0.65
18:0 Stearic	0.1	0.32	0.28	0.22
18:1 Oleic, cis	1.1	1.84	1.4	0.59
18:2 Linoleic, cis	2.6	4.24	1.46	0.65
18:3 Linolenic	0.3	0.64	3.44	0.08
Sat	0.7	1.2	0.69	5.61
MUFA	1.1	1.84	1.4	0.59
PUFA	2.9	4.88	4.9	0.73
n-3	0.3	0.64	3.44	0.08
n-6	2.6	4.24	1.46	0.65

Blank cells indicate no presence or insufficient data.

### O_3_ exposure

Animals were exposed to either filtered air (FA) or O_3_ for a single 4-h period via whole-body inhalation after 3 weeks of diet. O_3_ was generated using an OREC silent arc discharge generator (Osmonics, Phoenix, Arizona) as previously described (25). O_3_ concentration for the experiment was 1-part per million (PPM). This dosage has been utilized previously to produce a robust inflammatory response in the C57BL/6 model. Water was available for animals during the 4h exposure, but food was withheld. Exposures took place in standard shoebox cages without bedding (to prevent O_3_ scavenging).

### Bronchoalveolar lavage

Immediately following euthanasia (24 h following O_3_ exposure), BAL fluid was collected by tracheostomizing each mouse with a 20-gauge cannula. One milliliter of 1× Dulbecco’s phosphate-buffered saline (dPBS; Thermofisher Scientific) was inflated into the lungs and the suspension was retrieved from the lungs following inflation using a 1 ml syringe. The total number of viable white blood cells in the BALF was quantified using trypan blue-exclusion dye and a hemocytometer. Neutrophil and macrophage differentials were quantified from histology slides stained with hematoxylin and immunophenotyped as previously described (26). Following BAL collection, lungs were perfused with ice-cold PBS before being excised and snap frozen for lipidomic analysis.

### Untargeted lipidomics of mouse lungs

#### Reagents

HPLC-grade acetonitrile and methanol were purchased from Fisher Scientific. Phosphate buffer solution (PBS) and methyl tert-butyl ether (MTBE) were purchased from Sigma-Aldrich. HPLC-grade Chloroform was obtained from VWR. Standard compounds were purchased from Fisher Scientific, Sigma-Aldrich, and Avanti Polar Lipids in order to confirm detected lipids. PC (17:0/17:0) and PG (17:0/17:0) served as internal standards.

#### Sample preparation

Approximately 20 mg of lung tissue was mixed with 200 μl 10x-diluted PBS (4°C) and 80 μl internal standard solution [PC (17:0/17:0) and PG (17:0/17:0) in methanol; 50 μM; 4°C] in an Eppendorf tube (1.5 ml). Then stainless beads were added into the tube, and homogenization (2 min) was performed using a Bullet Blender homogenizer (Next Advance). After homogenization, 400 μl MTBE were added into the sample. Then the sample was vortexed for 30 s, stored under −20°C for 30 min, and sonicated in an ice bath for 10 min. After centrifugation (14,000 rpm, 10 min), 500 μl upper MTBE layer was collected into a new Eppendorf tube. The MTBE layer was then dried in a Vacufuge Plus Evaporator. Samples were then reconstituted with 100 μl 1:1 CHCl3: methanol. 80 μl of each sample was transferred to a liquid chromatography-mass spectrometry (LC-MS) vial for analysis, while the remaining 20 μl was pooled to create a quality control sample. The quality control step was measured every 10 samples to ensure consistent output by the LC-MS/MS.

#### Untargeted LC-MS lipidomics

The untargeted LC-MS lipidomics method used was similar to that previously reported (10, 11, 27). All LC-MS experiments were conducted using a Thermo Vanquish UPLC-Exploris 240 Orbitrap MS system. Each sample was run twice; one for positive ion mode and one for negative ion mode. For positive mode, 4 μl was used per injection, whereas 6 μl was used in negative ion mode injections. Both modes used reverse phase chromatography with a Waters XSelect HSS T3 column (150 × 2.1 mm, 2.5 μm particle size; Waters Corporation, Milford, MA). Flow through the column was maintained at 0.3 ml/min. The mobile phase Solvent A was comprised of 10 mM ammonium acetate in 60% H_2_O/40% acetonitrile. Solvent B consisted of 10 mM ammonium acetate in 90% isopropyl alcohol/10% acetonitrile. An isocratic elution was used with 50% solvent B for 3 min before its percentage was gradually increased to 100% over the next 12 min. Following 10 min of continued 100% solvent B, at t = 25 min, the percent of B was decreased gradually back to 50% to prepare for the next sample injection. Using mass spectrometer equipped with an electrospray ionization (ESI) source, we will collect untargeted data from 100 to 2000 m/z.

To identify peaks from the MS spectra, we made extensive use of the in-house chemical standards (∼800 lipids), and in addition, we searched the resulting MS spectra against the Human Metabolome Database (HMDB) library, Lipidmap database, METLIN database, as well as commercial databases including mzCloud, Metabolika, and ChemSpider. The absolute intensity threshold for the MS data extraction was 1,000, and the mass accuracy limit was set to 5 ppm. Identifications and annotations used available data for retention time (RT), exact mass (MS), MS/MS fragmentation pattern, and isotopic pattern. We used the Thermo LipidSearch 4.2 software for untargeted lipidomics data processing. The untargeted data was processed by the software for peak picking, alignment, and normalization. To improve rigor, only the signals/peaks with CV < 20% across quality control (QC) pools, and the signals showing up in >80% of all the samples were included for further analysis.

### Untargeted metabolomics of mouse lungs

#### Reagents

Acetonitrile, methanol, ammonium acetate, and acetic acid, all LC-MS grade, were purchased from Fisher Scientific. Ammonium hydroxide was bought from Sigma-Aldrich. DI water was provided in-house by a Water Purification System from EMD Millipore (Billerica, MA). PBS was bought from GE Healthcare Life Sciences. The standard compounds corresponding to the measured metabolites were purchased from Sigma-Aldrich and Fisher Scientific.

#### Tissue preparation

Briefly, each tissue sample (∼20 mg) was homogenized in 200 μl methanol: PBS (4:1, v:v, containing 1,810.5 μM 13C3-lactate and 142 μM 13C5-glutamic Acid) in an Eppendorf tube using a Bullet Blender homogenizer (Next Advance). Then 800 μl methanol: PBS (4:1, v:v, containing 1,810.5 μM 13C3-lactate and 142 μM 13C5-glutamic Acid) was added, and after vortexing for 10 s, the samples were stored at −20°C for 30 min. The samples were then sonicated in an ice bath for 30 min. The samples were centrifuged at 14,000 RPM for 10 min (4°C), and 800 μl supernatant was transferred to a new Eppendorf tube. The samples were then dried under vacuum using a CentriVap Concentrator (Labconco). Prior to MS analysis, the obtained residue was reconstituted in 150 μl 40% PBS/60% acetonitrile. A quality control sample was pooled from all the study samples.

### Data analysis and statistics

Normalization and statistical analysis were performed using Metaboanalyst© software (28). Analysis of lipid and fatty acid classes, as well as 2-factor ANOVAs of specific lipid species, were analyzed and graphed using GraphPad Prism (GraphPad Prism, v9.5.1). Pathway analysis for lipid class changes was performed using the BioPAN analysis tool (29). Additional pathway analysis utilizing Lipid Ontology web-based enrichment analysis, using a one-way ANOVA F-test and normalizing signals as percentages to calculate local statistics (30, 31).

## Results

### Animal weights

**Fig. 1:**
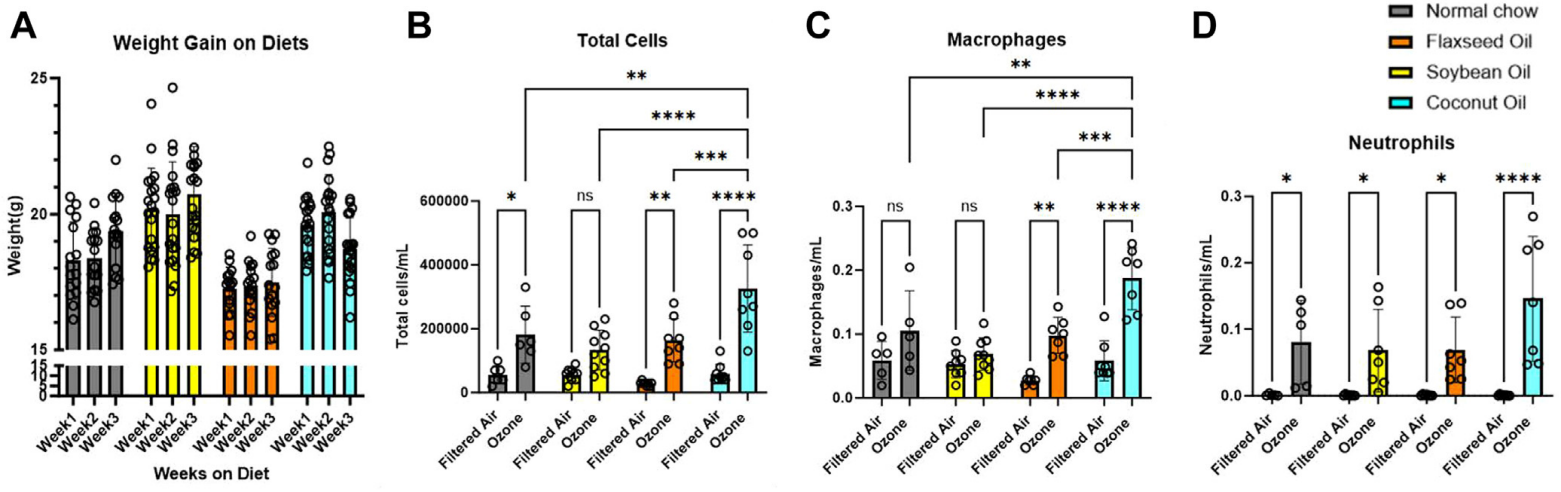
A: Weight gain for all dietary groups across 3 weeks of diet. B: Total cell counts from bronchoalveolar lavage 1-day post O_3_ exposure. C: Macrophage and (D) Neutrophil differential counts in lavage samples across groups. Data presented as mean with standard error. Asterisks indicate significant difference by two-way ANOVA from the indicated group, ∗*P* < 0.05; ∗∗*P* < 0.01; ∗∗∗*P* < 0.001; ∗∗∗∗*P* < 0.0001.

Animals were weighed weekly to assess differences in weight gain from the diets and as a measure of overall health. Animals in the SO and CO groups began the study with a modest increase in (<10%) starting weight when compared to the SC and FA groups. During the three-week experiment, animals showed no significant difference in weight gain between any of the dietary groups. The animals on the CO diet had a nonsignificant reduction in body weight between the second and third week on the diet (Fig. 1A).

### Bronchoalveolar lavage

Differential counts from BAL fluid samples showed characteristic increases in immune cell populations at 24 h following O_3_ treatment (Fig. 1B–D). Neutrophil and macrophage counts were higher all in the O_3_ exposed groups when compared to the filtered air controls of the same diet, this increase was statistically significant for the FA and CO dietary groups by way of 2-way ANOVA. The extent of the cellular infiltration in the CO diet group after O_3_ exposure was more pronounced than in the other dietary conditions, being significantly greater than the O_3_ groups of all three other diets. These data suggest greater inflammation in the animals in the dietary group containing higher saturated fatty acids in the supplemented oil. There was no significant difference in BAL cells between the diets in the filtered air groups, suggesting that none of the diets increased baseline immune infiltration to the lung.

### Dietary impacts on lung lipidomics

**Fig. 2:**
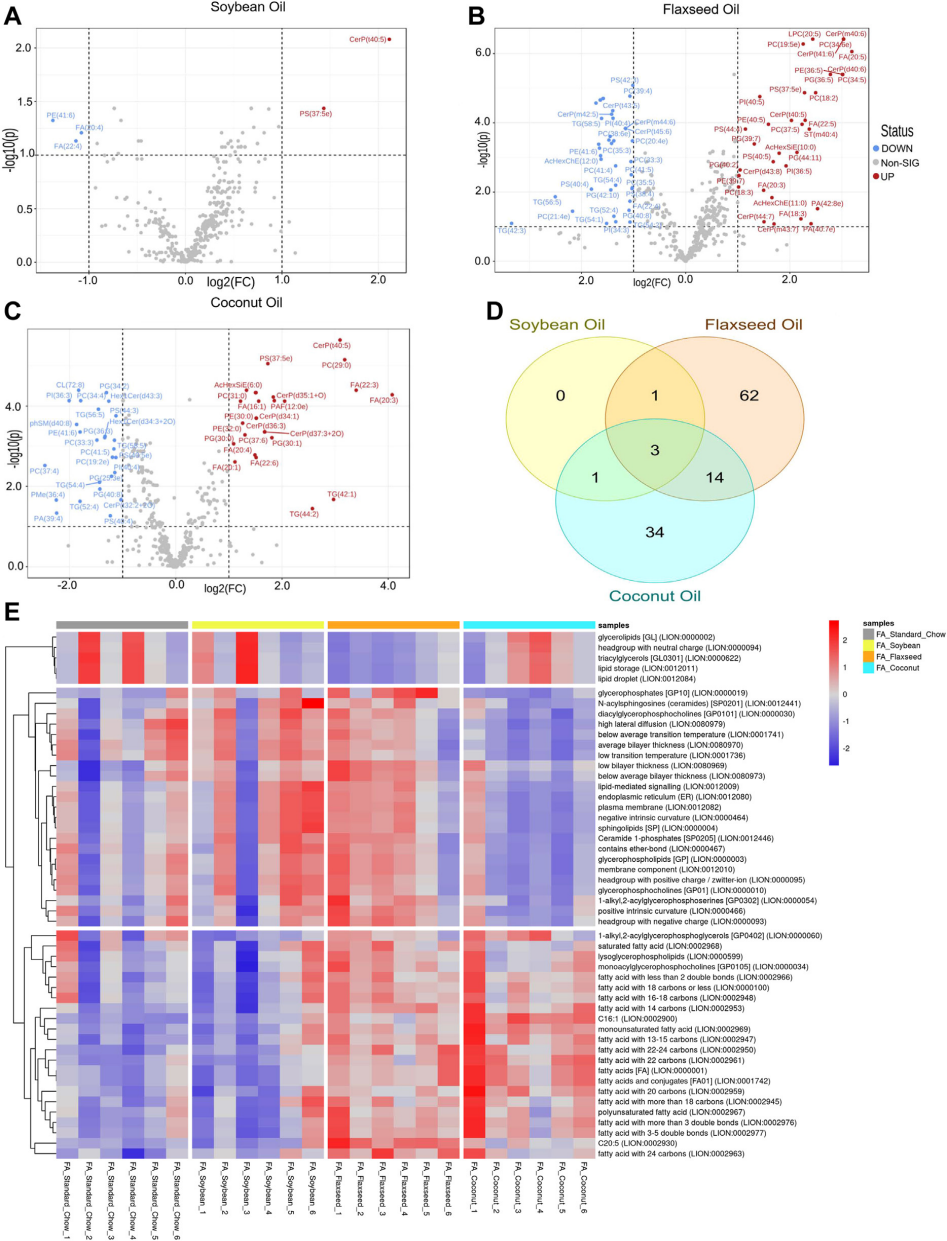
Lipidomic analysis of differences in diets in filtered air groups. Volcano plots depictions of comparisons between the differentially expressed lipid species in the standard chow diet and the (A) soybean oil diet, (B) flaxseed oil diet, and (C) coconut oil diet. D: Venn diagram showing overlapped lipid species differentially expressed in each diet compared with standard chow control. E: Lipid Ontology heatmap of Filtered air samples across the different dietary conditions.

Following sample preparation and mass spectrometry, we identified 483 lipid species that passed quality controls and were included in lipidomic analysis. As an initial approach, comparisons of each diet in the FA groups were conducted against the non-supplemented standard chow diet to see the effects of each diet on the lipidomic profiles of the lung. When analyzed by Student’s *t* test using an FDR cutoff of 0.1, the SO diet was most similar to the SC, with only two lipid species being significantly increased and three being significantly decreased (Fig. 2A). The FO diet had the largest change from SC, with 42 downregulated and 36 upregulated lipid species (Fig. 2B). The CO diet with the saturated fatty acids had an intermediate change, with 27 downregulated and 26 upregulated lipid species when compared to the standard chow diet (Fig. 2C). Of these differentially expressed lipid species, 3 were common amongst all three supplemented diets: CerP(t40:5), PE(41:6), and PS(37:5e) (Fig. 2D).

To gain a better understanding of the molecular processes being altered by diet, the dataset was analyzed through the lipid ontology (LION) bioinformatics tool (30, 31). A heatmap (Fig. 2E) of significantly altered lipid pathways provides biologically relevant molecular processes being affected by the change in diet. In agreement with the other analyses, a large proportion of lipidomic pathways being effected by the FO and CO diets were linked to fatty acids and fatty acid processing, such as polyunsaturated fatty acids (LION:0002967), fatty acids with 19–21 carbons (LION:0002949), and fatty acids and conjugates (LION:0001742).

**Fig. 3:**
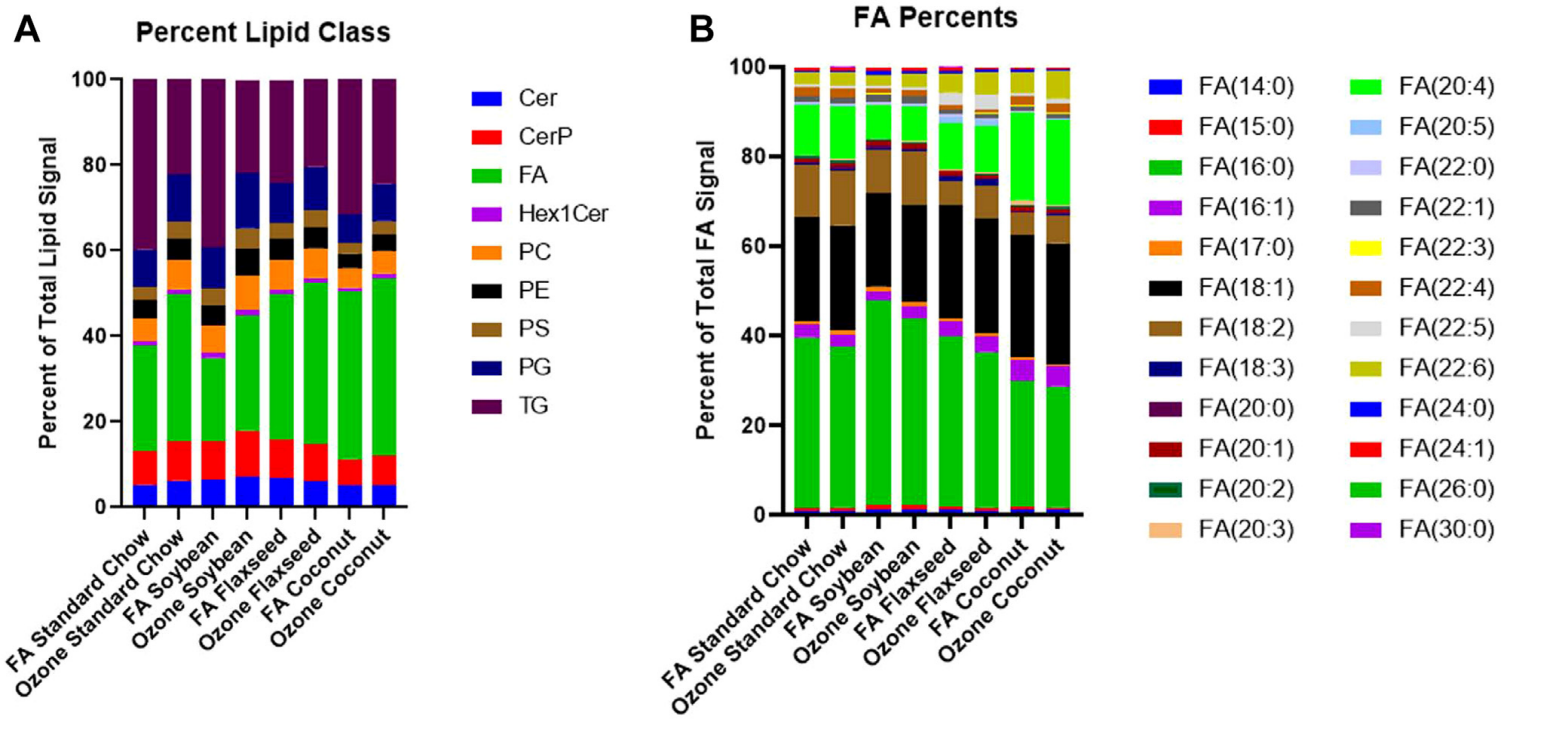
A: Proportions of the 9 most abundant classes of lipids identified in the dataset across dietary and exposure groups. B: Proportions of fatty acid species attributing to the total FA signal. Cer, Ceramides; CerP, Phosphoceramides; FA, Fatty Acids; Hex1Cer, Hexosylceramides; PC Phosphocholines; PG, Phosphoglycerols; PE, Phosphoethanolamines; PS, Phosphoserines; TG, Triacylglycerols.

To gain a more generalized idea of how the complex lung lipidome may be altered, the seven most abundant classes of lipid species were separated and compared. When comparing the total lipid signal of these seven classes, the SC and SO diets had a lower proportion of fatty acids and a higher proportion of triacyglycerols than the FO or CO-supplemented diets in the filtered air groups (Fig. 3A). When analyzed separately, the individual fatty acids contributing to the total FA signal for each condition reveal palmitic acid (FA16:0) and vaccenic acid (FA18:1) to be the major contributing FAs to the total FA signal. The SO diet had the highest percent of total palmitic acid signal (47.75%) and the coconut oil diet had the lowest percent of palmitic acid (29.93%). Palmitic acid is the most abundant fatty acid in the body, accounting for between 20%-30% total fatty acids in humans (32). High fat diets rich in palmitic acid have been shown experimentally to exacerbate pulmonary fibrosis following bleomycin induced lung injury (33). The CO diet also had the highest percent of eicosatetraenoic acid (FA20:4) (Fig. 3B).

**Fig. 4:**
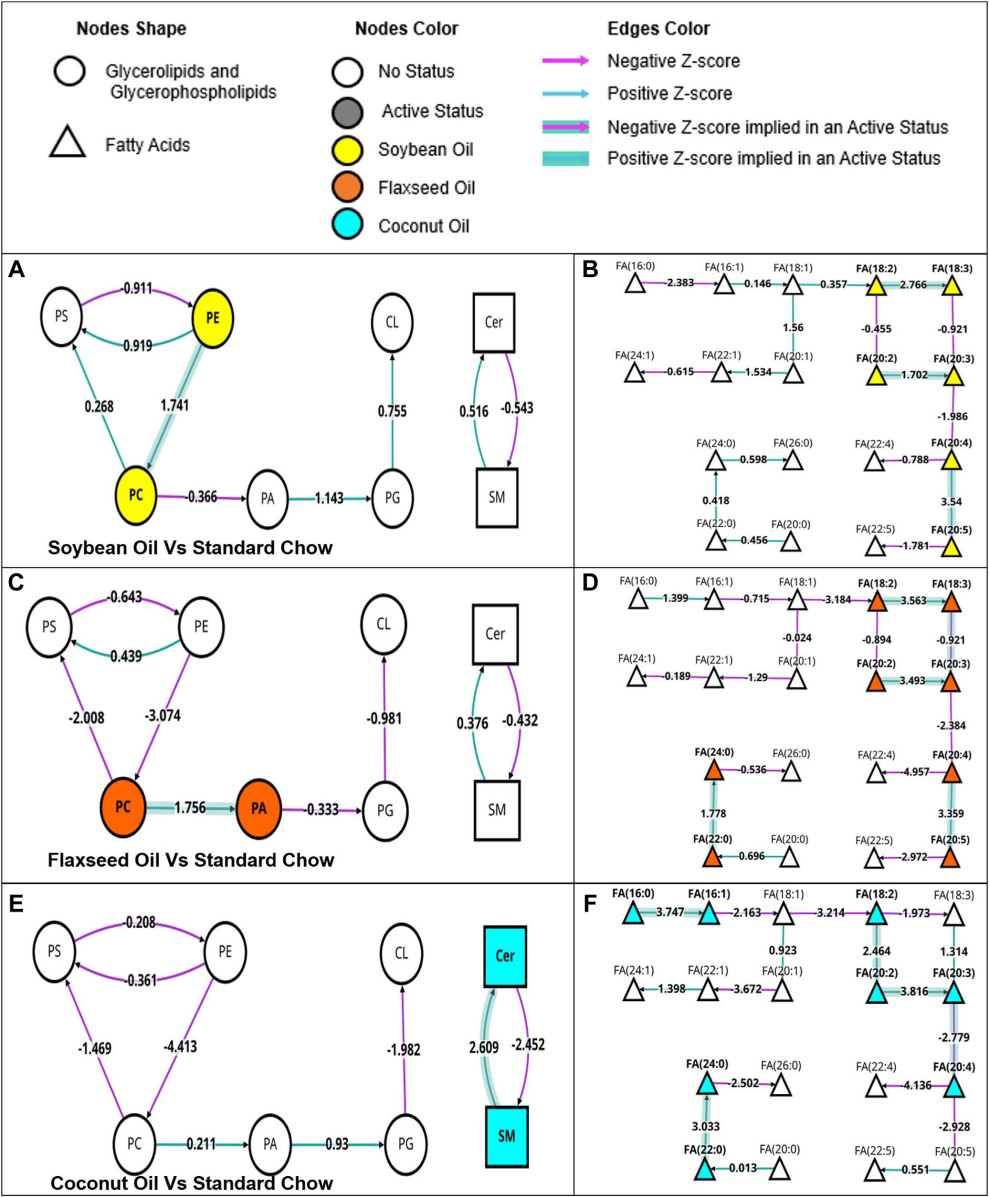
BioPAN lipid class analysis of diet effects as compared to standard chow diet. Active nodes represent activated lipids/lipid classes with colors specific to each individual diet (yellow = soybean oil, orange = flaxseed oil, blue = coconut oil), with Z scores detailing implied conversions contributing to activated states. A: Overall lipid class changes when comparing the soybean oil-enriched diet to the standard chow diet for control animals. B: fatty acids change for the soybean oil diet against the standard chow diet. C: Overall lipid class changes when comparing the flaxseed oil-enriched diet to the standard chow diet for control animals. D: fatty acids changes for the soybean oil diet against the standard chow diet. E: Overall lipid class changes when comparing the flaxseed oil-enriched diet to the standard chow diet for control animals. F: fatty acids changes for the soybean oil diet against the standard chow diet. Cer, ceramide; CL, cardiolipin; PA, glycerophosphate; PC, phosphocholine; PE, phosphoethanolamine; PG, phosphoglycerol; PS, phosphoserine; SM, sphingomyelin.

The dataset was then analyzed through BioPAN® software to examine interactions between lipid types. The dietary effects or each oil-supplemented diet when compared to the standard chow control are outlined in Fig. 4. The web-based tool was able to sort individual lipid species into 8 classes of lipids [phosphoserines (PS), phosphoethanolamines (PE), phosphocholines (PC), glycerophosphates (PA), phosphoglycerols (PG), cardiolipins (CL), ceramides (Cer), and sphingomyelins (SM)]. Interactions between the different classes are mapped, and activated statuses are highlighted. The SO supplemented diet was shown to activate PE and PC classes, with an implied contribution of PE to PC (Fig. 4A). The FO supplemented diet had an activation and transition of PCs to PAs (Fig. 4C), while the coconut oil diet displayed and activation and transition of SMs to Cers (Fig. 4E). When examining fatty acid species, both of the PUFA supplemented SO and FO showed activation of linolenic acid (FA18:3) along the pathway to the activation of eicosapentanoic acid (EPA, FA20:5; Fig. 4B, D). The CO diet showed activation of linoleic acid (FA18:2) to eicosatrienoic acid (FA20:3), as well as palmitic acid (16:0) to palmitoleic acid (FA16:1; Fig. 4F).

#### Ozone-induced changes to lung lipid profiles

The O_3_ effects on lipid composition within each dietary group were then analyzed by comparing to the filtered air groups as controls. Following O_3_ exposure the proportions of fatty acids increased in the SC and SO groups, with a concomitant reduction in TGs. The FO- and CO-supplemented groups did not show major changes in the proportions of lipid classes following O_3_ exposure (Fig. 3A).

**Fig. 5:**
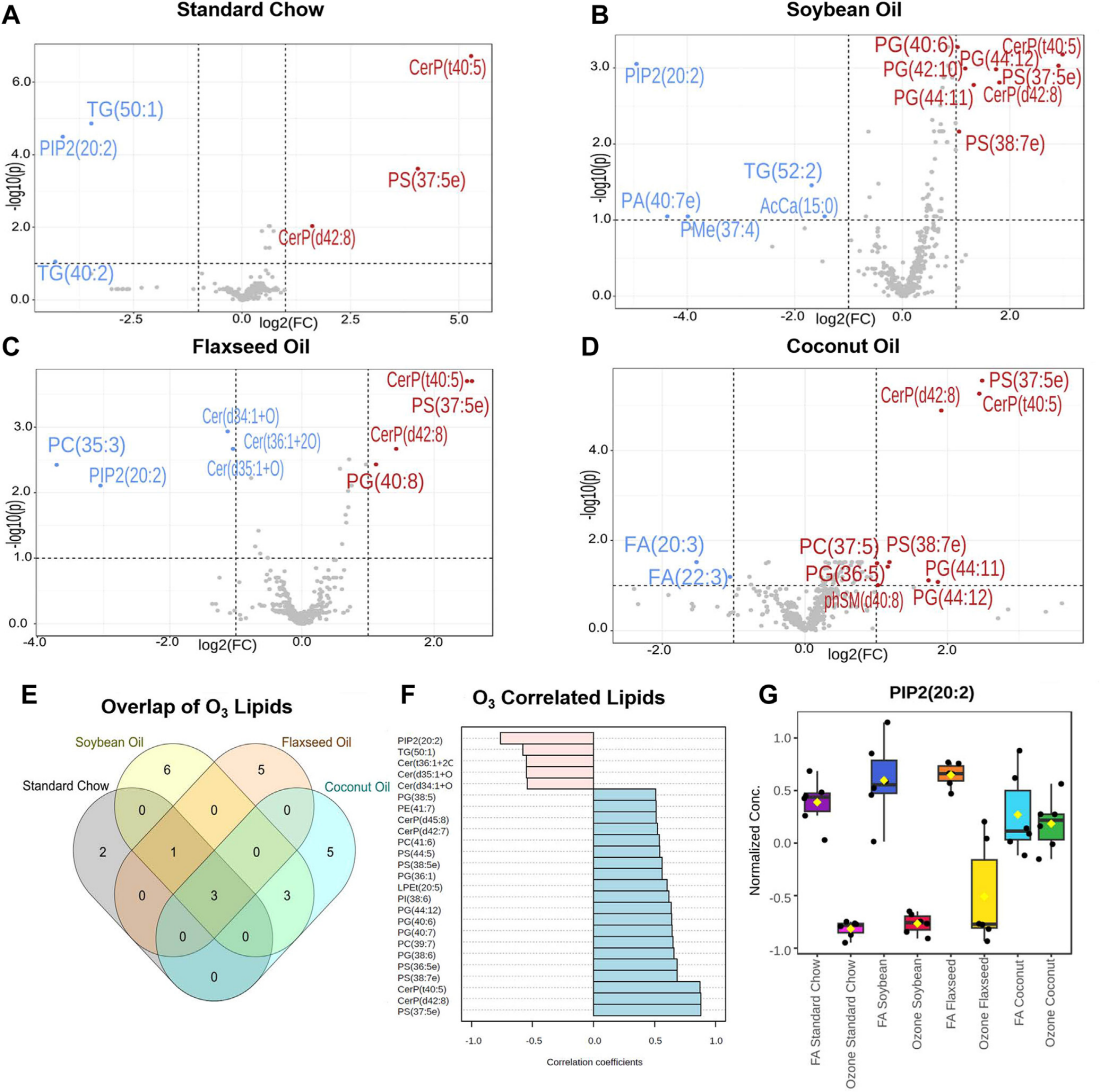
Volcano plots depicting lipidomic changes following O_3_ exposure in (A) Standard Chow group, (B) Soybean Oil group, (C) Flaxseed Oil group, and (D) Coconut Oil group. (E) Venn diagram depicting the overlap of significantly altered lipids between diets. (F) Metanalysis of top 25 lipid species associated with Exposure. (G) Concentrations of PIP2(20:2) across all dietary/exposure conditions.

Across our panel of lipids, the effect of O_3_ exposure on the overall lipid composition of the lung was modest. There were six lipids significantly altered by exposure in the SC group, thirteen in the SO group, nine in the FO group, and nine in the CO group (Fig. 5A–D). All four dietary groups consistently upregulated the expression of CerP(d42:8), CerP(t40:5), and PS(37:5e) in response to O_3_. Meta-analysis of the 25 top lipids correlated with exposure identified PIP2(20:2) to be highly correlated with exposure (Fig. 5F). Interestingly, PIP2(20:2) is downregulated in response to O_3_ exposure in the SC, SO, and FO groups, but is not significantly altered in the CO group which had the greatest cellular effect from O_3_ via BAL analysis. PIP2(20:2) is a phosphtidylinositolbisphosphate, containing a palmitic acid moiety as well as an eicosadienoic acid (EDA) moiety. EDA can be metabolized to form dihomo-γ-linolenic acid and subsequently arachidonic acid (34).

**Fig. 6:**
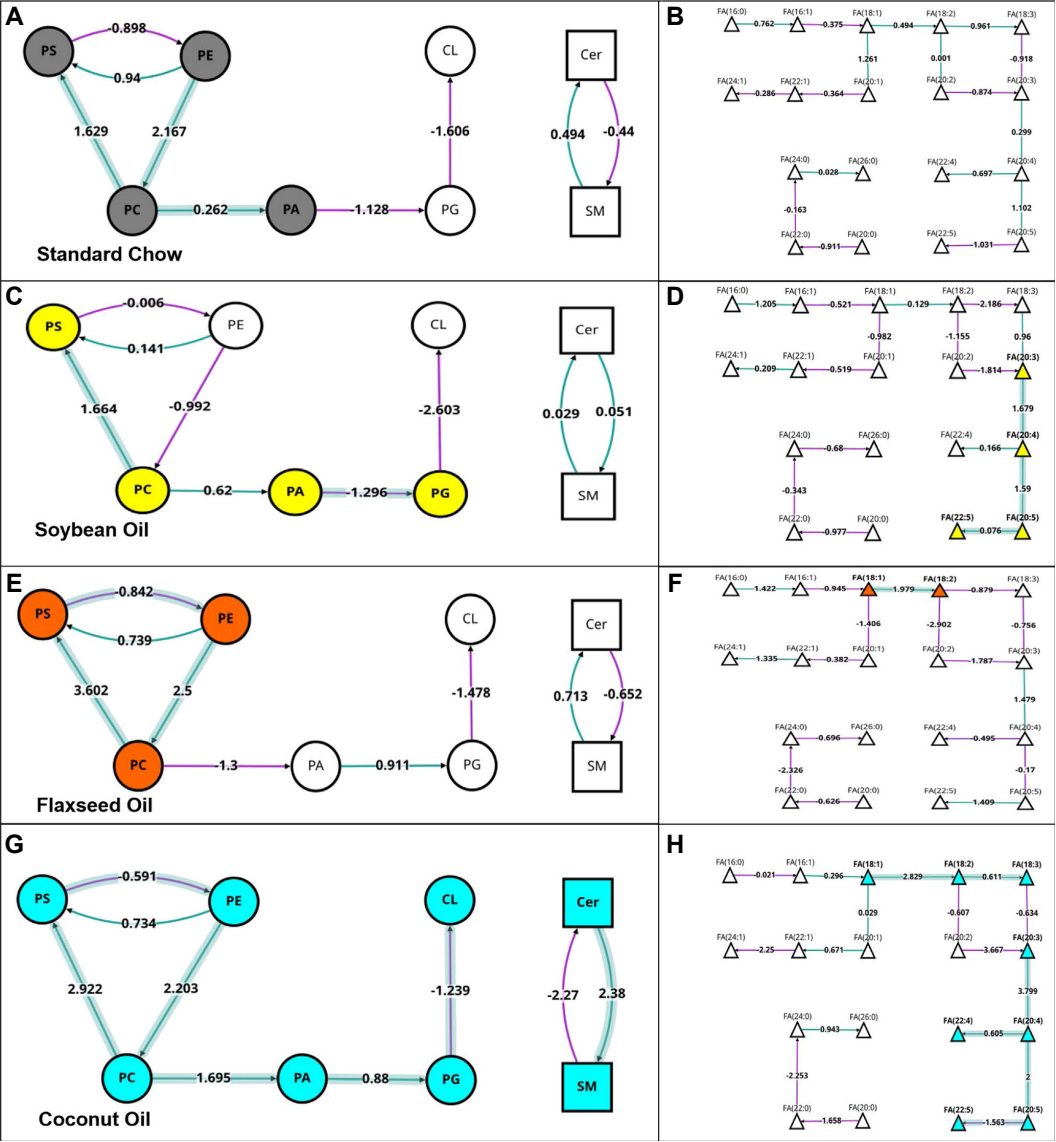
BioPAN lipid analysis of lipid class (left column) and fatty acids (right column) from lungs of animals fed standard chow (A, B), soybean oil (C, D), flaxseed oil (E, F), or coconut oil (G, H). Highlighted nodes indicate significant activation of lipid classes/fatty acids in O_3_-exposed animals as compared to filtered air controls.

Analysis of the O_3_ effects by BioPAN showed more robust activation profiles of lipid classes. The SC diet showed active status for PS, PE, PC, and PA class lipids, the SO-supplemented diet showed active status of PS, PC, PA, and PG class lipids, and the FO-supplemented diet showed active status of PS, PE, and PC class lipids. The CO-supplemented diet showed an O_3_ effect with active status for all 8 lipid classes analyzed (Fig. 6A, C, E, G). The fatty acid pathways showed fewer overall changes due to O_3_ exposure. The SC diet group did not display any active status in response to O_3_ for any of the fatty acid species presented in this analysis (Fig. 6B). The SO supplemented diet showed activation of fatty acids among the eicosanoids including FA(20:3), FA(20:4), FA(20:5) and FA(22:5) (Fig. 6D). The FO supplemented diet showed activation of FA(18:1) and FA(18:2) (Fig. 6F). The CO supplemented diet had the greatest active statuses among fatty acids in response to O_3_, showing similar activation of FA(18:1) and FA(18:2) to the FO group, as well as FA(18:3). The CO supplemented diet also showed active status in the same eicosanoids as the SO diet, with the addition of FA(22:4) (Fig. 6H). This analysis indicates the CO diet had a more robust, global reaction to the O3 exposure in all pulmonary lipid classes than the PUFA supplemented or SC diets. A table of reaction chains, and predicted genes associated with these lipid conversions (Table 2), shows the CO group having the most diverse set of transitions being predicted, with the SO group having the least in response to O_3_ inhalation.

**Table 2:** significant lipid species reactions and predicted genes for facilitating molecular transitions for respective pathways

Reactions Chains	Z-score	Predicted Genes
Standard Chow Ozone Effects		
PE→PC→PS	2.684	*PEMT, PTDSS1*
PE→PC→PA	1.718	*PEMT, PLD1, PLD2*
Soybean Oil Ozone Effects		
PA→PG	1.872	*CDS1, CDS2, PTPMT1*
Flaxseed Oil Ozone Effects		
PE→PC→PS	4.315	*PEMT, PTDSS1*
PC→PS→PE	1.952	*PTDSS1, PISD*
Coconut Oil Ozone Effects		
PE→PC→PS	3.624	*PEMT, PTDSS1*
Cer→SM	2.38	*SGMS1, SGMS2, CERT1*
PC→PA→PG	1.821	*PLD1, PLD2, CDS1, CDS2, PTPMT1*
PE→PC→PA→PG→CL	1.769	*PEMT, PLD1, PLD2, CDS1, CDS2, PTPMT1, CRLS1*
PC→PS→PE	1.648	*PTDSS1, PISD*

**Fig. 7:**
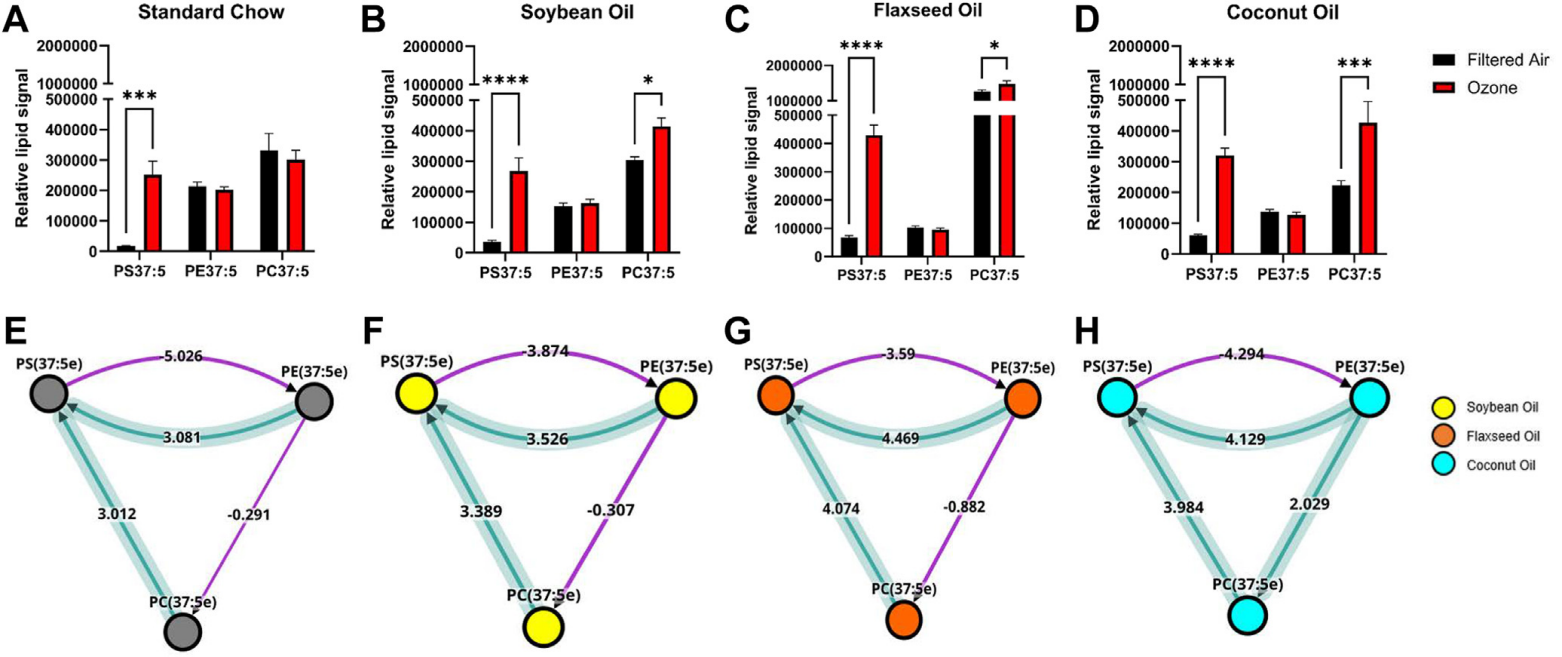
Bar graphs of PE(37:5e) (E), (PS37:5e) (F), and PC(37:5) (G) showing significant comparisons by Two-Way ANOVA(A–D). BioPAN lipid analysis of O_3_-specific effects between (PS37:5e), PE(37:5e), and PC(37:5e) (E–H). Asterisks indicate significant difference from indicated group, ∗*P* < 0.05; ∗∗*P* < 0.01; ∗∗∗*P* < 0.001; ∗∗∗∗*P* < 0.0001 by Two-way ANOVA.

In addition to overall lipid classes and fatty acids, the O_3_-specific effects in lipid species were also analyzed via BioPAN. A complete map and table of lipid species interactions can be found in the supplement. Of particular interest were the interactions between PS(37:5e), PE(37:5e), and PC(37:5e). All three of these lipid species showed active status, and implied interactions from both PE(37:5e) and PC(37:5e) to PS(37:5e) in response to O_3_ in all dietary conditions (Fig. 7E–H). PS(37:5e) was the most significantly associated lipid with the O_3_ exposure, being significantly upregulated in under all dietary conditions (Fig. 7A–D), with the most robust upregulation occurring in the FO supplemented diet (Fig. 7C).

**Fig. 8:**
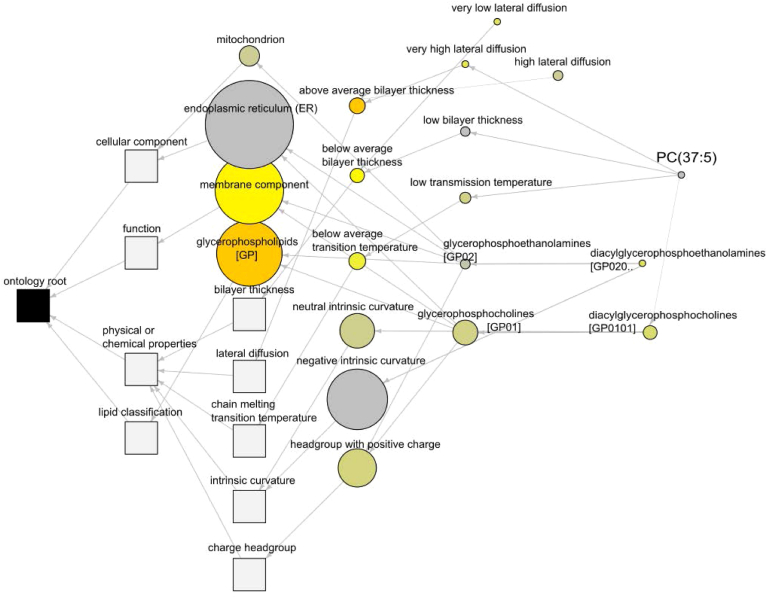
Lipid Ontology (LION®) pathway analysis of total lung lipids. Network of lipidomic processes influenced in the dataset stemming from interactions with PC(37:5e).

**Fig. 9:**
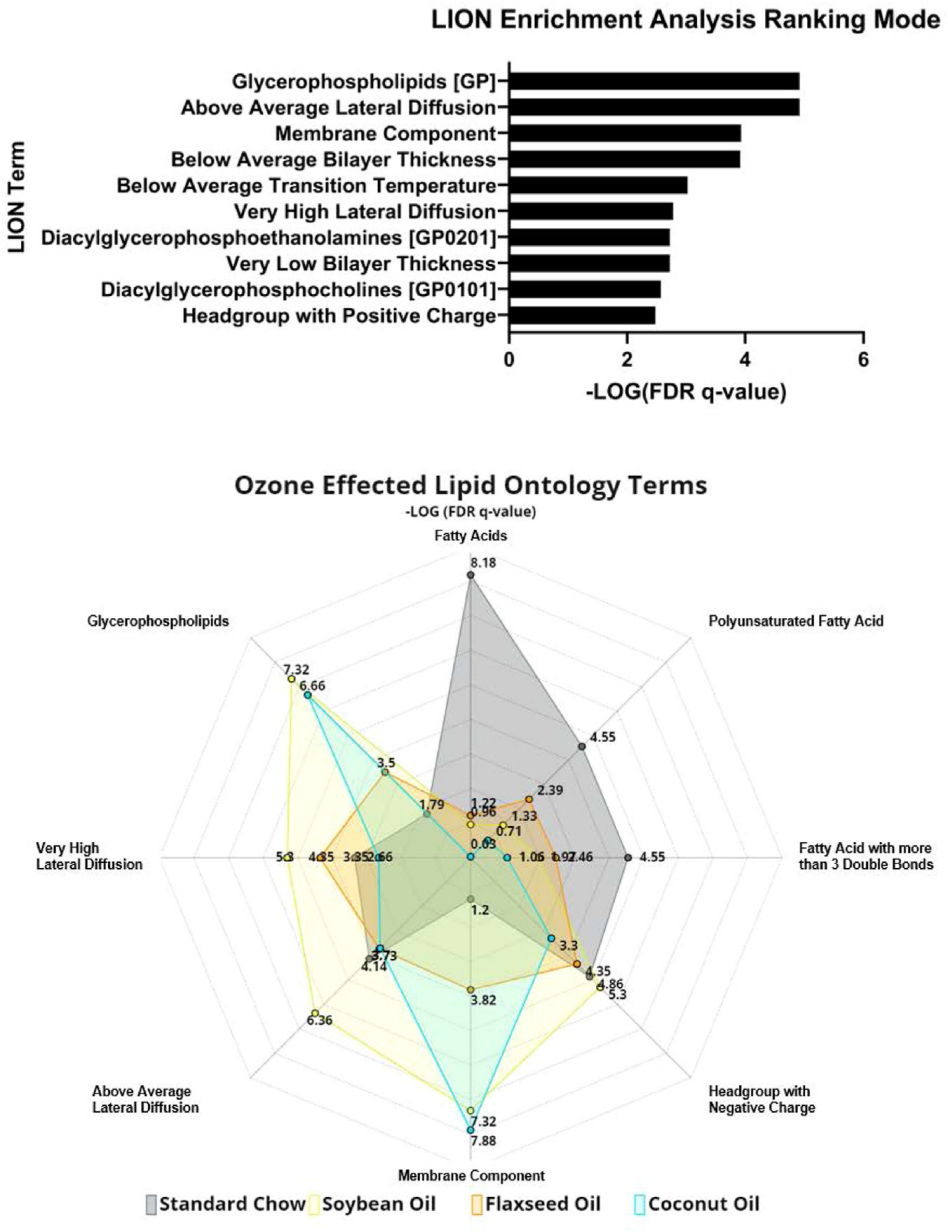
Bar graph (TOP) of significant lipidomic processes highlighted in Lipid Ontology analysis, with Glycerophospholipids and above-average lateral diffusion being the most significant processes, corresponding to highlighted nodes in the previous figure. Radar plot (Bottom) of top 10 commonly ranked LION terms affected by ozone exposure in each diet.

**Fig. 10:**
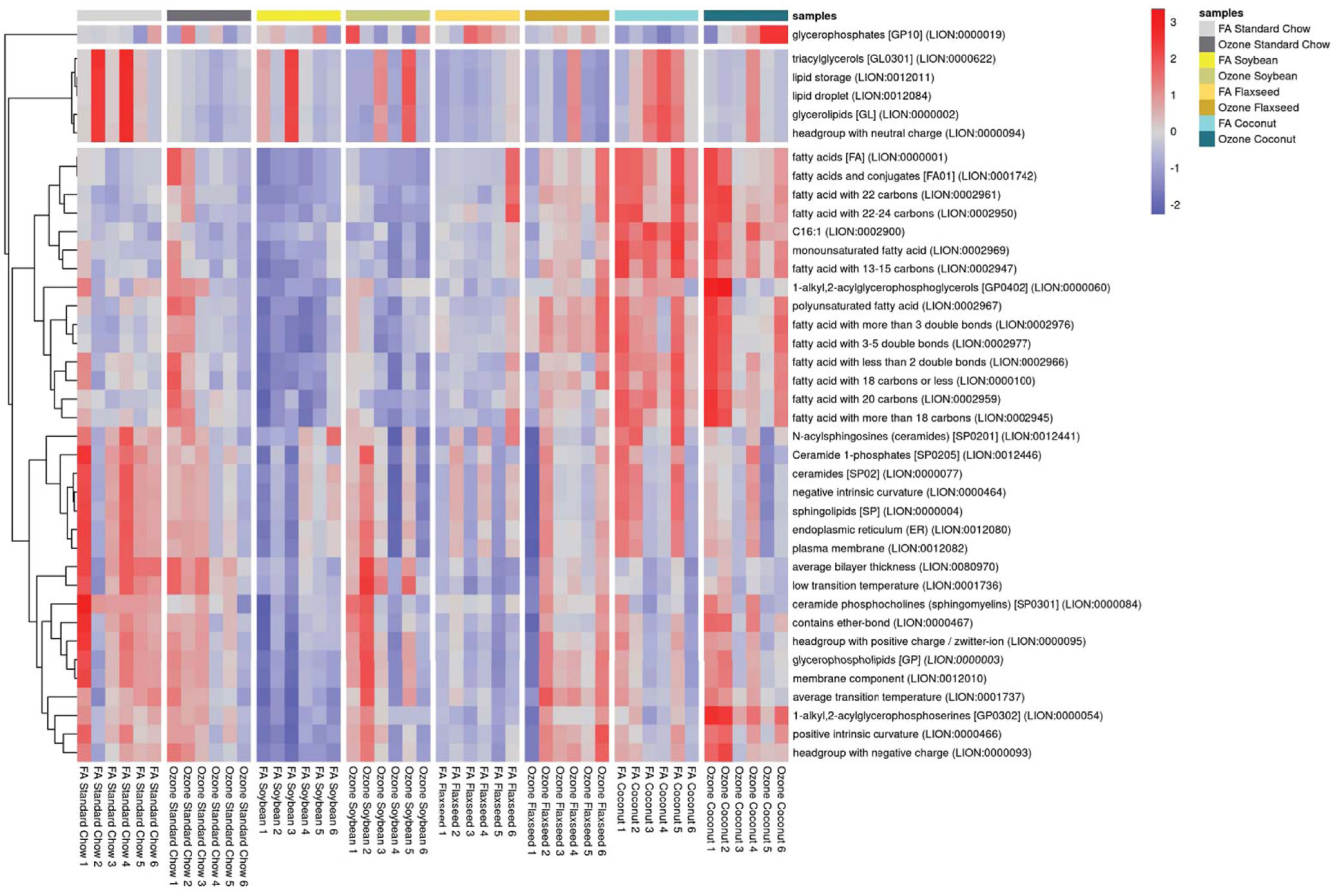
Heatmap of enriched terms across all samples from Lipid Ontology analysis.

Within the Lipid Ontology analysis, 425 out of 483 total lipids for the dataset (87.99%) amongst all groups were annotated to LION identifiers for pathway analysis. This analysis found glycerophospholipids (LION:0000003) and above average lateral diffusion (LION:0080982) to be the highest ranked terms from the dataset by one-way ANOVA (Fig. 8). When analyzed individually by Student’s *t* test, the ozone effects of each dietary group showed similar LION pathways, as shown in Supplemental Tables 1–4 The top 8 common LION pathways by exposure for each diet are outlined in a radar chart (Fig. 9). A heatmap of enriched terms for all groups (Fig. 10) shows visually apparent dietary effects of CO in membrane composition, and distinctions in fatty acid processing between the SC and SO supplemented diets when compared to the FO and CO supplemented diets. Of particular interest, C16:0 and C16:1 (LION:002882, LION:0002900) were both significantly enriched specifically in the CO group. This interaction of palmitic acid (16:0) and palmitoleic acid (16:1) and its positional isomers as highlighted in the BioPAN analysis (Fig. 4F) may provide useful information about the role of monounsaturated fatty acids (MUFAs) in the lung. Palmitoleic acid as a lipokine has been studied for its role in cardiovascular disease, diabetes, nonalcoholic fatty liver disease, as well as cancer (35).

**Fig. 11:**
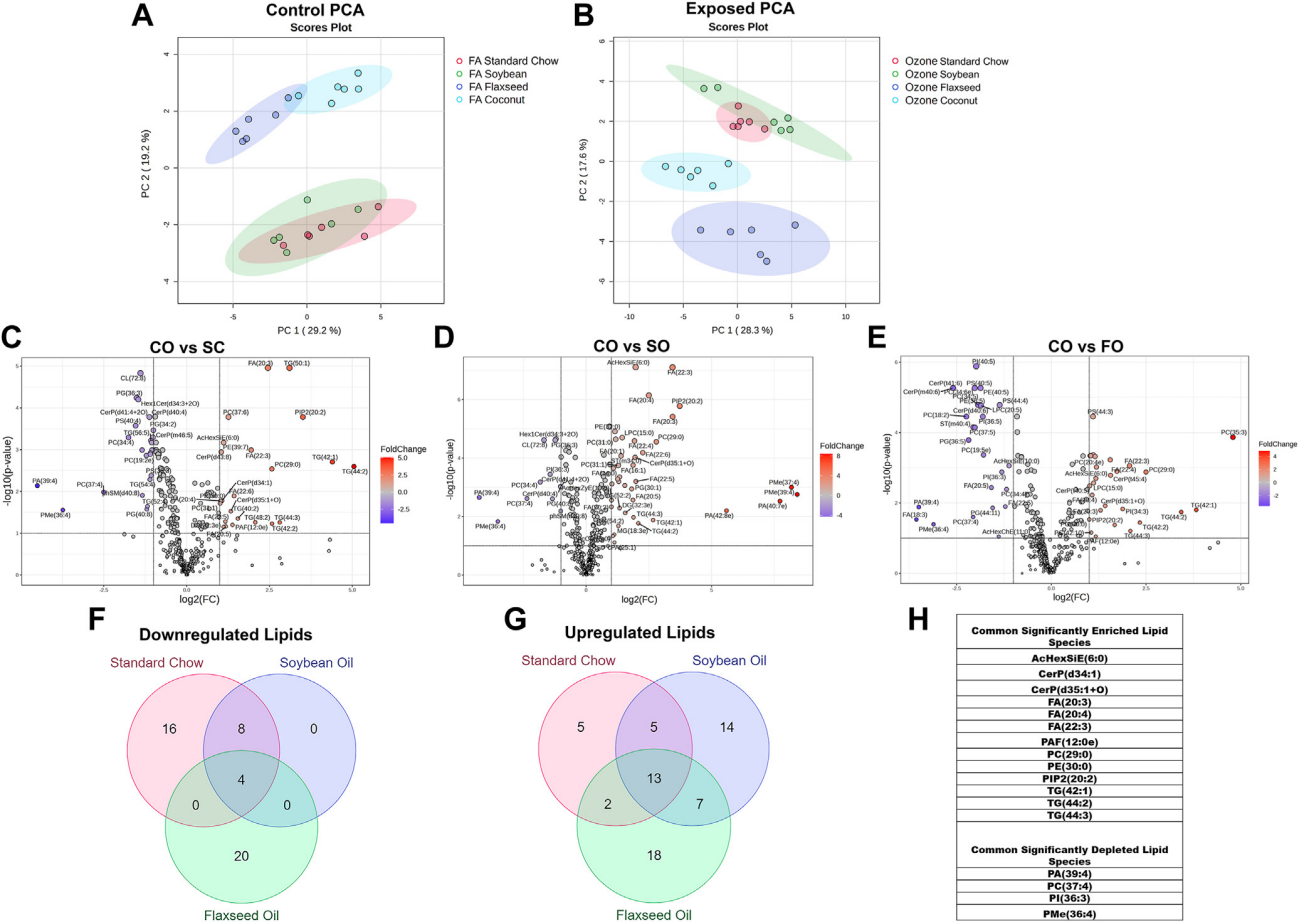
Principle component analysis (PCA) diagrams for filtered-air control (A) and ozone-exposed (B) animals. Volcano plots detailing differentially expressed lipids between the coconut oil diet and standard chow (C), soybean oil (D), and flaxseed oil (E) diets from animals exposed to O_3_. Venn diagrams of shared and unique differentially expressed lipid species across groups (F, G), with shared lipid species tabulated (H).

When comparing the O_3_-exposed groups as a whole, principle component analysis reveals greater separation of dietary groups when compared to the air control group (Fig. 11A, B). Due to the increased cellular influx observed in the BAL, the CO-O_3_ diet was compared against the other O_3_-exposed diets to examine specific changes in the lung microenvironment. The CO-O_3_ lungs had 53 significanty differentially expressed lipid species when compared to the SC-O_3_ diet, 51 compared to the SO-O_3_ diet, and 64 compared to the FO-O_3_ diet, respectively (Fig. 11C–E). Of these lipid specied there were 4 common downregulated lipids and 13 common upregulated lipids (Fig. 11F–H). Of note, FAs 20:3, 20:4, and 22:3 were enriched against all other O_3_-exposed groups, as well as PIP(20:2). These fatty acids may be unable to be processed into specialized pro-resolving lipid mediators (SPMs) to aid in the resolution of the O_3_-induced inflammation.

## Discussion

Dietary lipid modulation in the control group (FA) led to clear changes in the lung lipidomic profile following the 3-week regimen. The most significant changes included alterations in the concentrations of CerP(t40:5), PE(41:6), and PS(37:5e). PS(37.5e) was increased in each of the supplemented diets, and highly upregulated 24 h following ozone exposure. Similarly, following pulmonary insult by O_3_ dietary lipid modulation led to significant increases in CerP(t40:5) and CerP(d42:8) among all dietary groups and decreases in levels of PIP2(20:2) in all dietary groups with the exception of CO. Given the robust increase in cellular infiltration to the lung, it was concluded that CO supplementation created a more vulnerable environment for inflammatory responses and SO supplementation produced a reduced infiltration that failed to reach significance such as the levels of the other diets. Overall, the results of this study demonstrated a robust response to dietary lipid modulation and to dietary lipid modulation following O3 exposure on lipidomic profile and pathways. These findings provide critical insight for next level studies on lipid-mediated inflammatory effects and identification of therapeutic targets. Specifically, bronchoalveolar lavage showed characteristic increases in total infiltrating cells after exposure to O_3_, with a more pronounced infiltration in the animals receiving the CO diet after O_3_ exposure.

In terms of cellular microenvironments, the lungs represent a unique biological interface with ambient air for facilitating molecular interactions. This air-liquid interface provides an entry route for xenobiotic toxicants and volatile organic compounds that can lead to inflammation or, in extreme circumstances, direct damage to the alveolar regions or connecting airways. Cytotoxic and pro-inflammatory responses to ozone have been well documented in in vivo and in air-liquid interface cell culture (36). The lipid and protein compositions of the ELF of the lung largely dictate the extent of pulmonary inflammation and injury in the deep lung. Human ELF contains a diverse array of lipid species, with glycerophospholipids accounting for over 90% of the average abundance (10). Dietary supplementation of omega-3 fatty acids has increased in prevalence for a variety of health outcomes, including heart failure, arrhythmia, cardiomyopathy, and hypertension (37). The current study sought to identify how dietary supplementation of different saturated and polyunsaturated fatty acids might cause changes to the lipidomic profile of the lung, as well as the impacts on inflammation from O_3_ exposure. The data presented here utilize lung homogenates containing traces of ELF as well as bulk lung interstitium to generate pulmonary lipidomic samples. Future studies may benefit from a more targeted analysis of surfactant-specific lipids as a site of initial oxidative damage, whereas the data presented here reflect more global changes to the lung as a whole based on diet or exposure.

Our untargeted lipidomic analysis revealed modest changes in overall pulmonary lipids in our FO and CO diets when compared to a grain-based SC diet. Following O_3_ exposure, we found the expression of CerP(d42:8), CerP(t40:5), and PS(37:5e) to be increased in all of our dietary conditions. Studies of hyperoxic acute lung injury have shown PS and cardiolipin to increase in the mouse lung following oxidative stress (38). Cardiolipins were significantly activated in the CO group in response to ozone (Fig. 6G) We found PIP2(20:2) to be decreased in three of our dietary conditions, but not in the CO diet. Effects of PIP2(20:2) on O_3_ induced lung inflammation have yet to be studied in detail, providing a potentially useful molecular target for future studies. Mechanistically, ozone has been shown previously in in-vivo studies to be involved in PI3K/Akt/NF-kB signaling pathway (39, 40, 41). Palmitic acid, such as that found in high abundance in coconut oil, has been shown to suppress the PI3K/Akt pathway (42). As a source of cellular PIP2, the changes in PIP2(20:2) levels observed in the current study may be modulating downstream inflammatory processes of PI3K.

Lipid ontology pathway analysis revealed glycerophospholipids and lateral diffusion to be significantly affected in our study. Lateral diffusion, the process of lateral movement of lipids or proteins across the inner or outer portion of the lipid membrane, is a fundamental property of membrane integrity, where interactions with membrane-bound proteins influence cellular function and response to stimuli (43). Specifically, animals in the SC diet group had more robust upregulations in fatty acids, whereas animals fed the SO diet showed increases in lateral diffusion, a mechanism that may influence the reduced cellular influx in BAL fluid. More targeted approaches will be needed to characterize how the pulmonary lipid organization influences responses to ozone and other airborne toxicants.

Recent studies utilizing O_3_ as a pulmonary toxicant have shown sex differences in responses of pulmonary eicosanoids and SPMs in mouse lungs. Specifically, female mice had significantly higher levels of several eicosanoid species 24 h after O_3_ exposure when compared to male mice (44, 45). SPMs have been implicated in the resolution of inflammation from air pollutants such as occupational dusts and fumes, allergens, and tobacco smoke, and their therapeutic potential is being investigated for many respiratory diseases caused by chronic lung inflammation such as COPD, pulmonary fibrosis, as well as lung cancer (46, 47). While the untargeted lipidomic approach employed in this study did not measure specific SPMs, the dietary influences of PUFA and saturated fatty acid supplemented diets on pulmonary eicosanoids shown here may be relevant to more targeted future analyses on diet and SPM function. As an important source of short-chain fatty acids (SCFAs), the fermentation of dietary fibers plays a crucial role for production of precursory FA moieties to drive not only metabolism but pro or anti-inflammatory processes (48). As shown in Fig. 9, the grain-based SC diet had altered FA pathways when compared against the enriched diets. While the grain-based and enriched diets as a whole did not significantly affect weight gain or characteristic ozone-induced neutrophilia (Fig. 1), much of the lipidomic analysis comparing the grain-based SC diet against the supplemented enriched diets has the potential to confound the interpretation of the data (49). The FO and CO diets are alterations of the SO, serving as an internal control to mitigate some of the issues with comparing grain-based and enriched diets.

Other limitations of this study include the inability to address oxidized forms of lipids, as well as the potential for protein adducts from the oxidized lipid intermediates. O_3_ is known to interact with ELF phospholipids, antioxidants (*eg*, urate, glutathione, ascorbate), and surfactant proteins (50). We established that dietary lipid changes alter the initial conditions for the O_3_ reactions, and ultimately modifies the inflammatory response. However, whether the benefits of dietary lipids are due to initial O_3_ reactions with ELF phospholipids or instead due to mitigation of immune cell responses is not discernible from this study design. Another limitation of our study was that we did not differentiate the lipid responses of the ELF specifically, compared to the whole lung, and we postulate that the proximal O3-ELF interaction effects are greatly diluted in the whole lung homogenate. Lung samples in the current study underwent a bronchoalveolar lavage prior to lipidomic processing. This process may remove ELF lipid mediators, and future studies may benefit from analyzing these alveolar microenvironments in a more targeted manner. Furthermore, the time frame of examining lipid changes 24h post exposure allows limited insight into the immediate, rapid oxidative lipid changes at this air-liquid interface during exposure. Scientific inquiry at multiple time points is necessary to fully appreciate the complex sequelae of molecular and cellular responses in the lung microenvironment in terms of both damage and resolution. Despite having no changes in weight gain over the course of the study between groups, there are slight differences in total weight between groups. While we do not anticipate these differences causing significant changes to pulmonary lipid profiles, it is documented that obesity can modify responses to ozone (51, 52), and this should be taken into consideration when interpreting these results. Finally as alluded to above, we did not investigate the role of sex and dietary modifications. Additional investigation would help clarify molecular mechanisms of protection/susceptibility and provide more targeted approaches to potential environmental health interventions.

In summary, air pollutants like O_3_ have broad health impacts and advisory statements are typically limited to avoidance measures, as pharmacotherapeutic strategies are not practically or standardly utilized to address public health concerns. Dietary recommendations may be a more plausible approach to help offset the deleterious effects of environmental exposures in large populations. The present study, with limitations of the research model and study design, provides important context for the basis of how altered dietary lipids can alter the lung microenvironment to make individuals more or less susceptible to external stressors, as evidenced by the more robust cellular infiltration in the CO group following O_3_ exposure. The diet-induced exacerbation in ozone-induced cellular inflammation shows the potential negative effects of excess oil in the diet. Future studies that define the breadth of potential benefits from dietary lipids or the reduction of problematic dietary lipids in concert with antioxidants, in addition to exploring other air pollutants, would further refine such recommendations.

## Data availability

Supplemental and raw data files are available via the NIH metabolomics workbench.

## Supplemental data

This article contains supplemental data.

## Conflict of interst

The authors declare the following financial interests/personal relationships which may be considered as potential competing interests.

Matthew Campen reports financial support was provided by National Institutes of Health. If there are other authors, they declare that they have no known competing financial interests or personal relationships that could have appeared to influence the work reported in this paper.
