# Supplementary material for: Dietary modulation of lung lipids influences inflammatory responses to inhaled ozone
Source: J Lipid Res. 2024 Aug 30;65(9):100630. doi: 10.1016/j.jlr.2024.100630 (PMC11417538; doi:10.1016/j.jlr.2024.100630)
Supplement: Supplementary Tables [file mmc1.docx]

Data Supplement to: **Dietary modulation of Lung Lipids Influences Inflammatory Responses to Inhaled Ozone**

**Russell Hunter^1^, Brenna Baird^1^, Milad Mazloumi-Bakhshayesh^1^, Siem Goitom^1^, Selita Lucas^1^, Guy Herbert^1^, David Scieszka^1^, Edward Davis^2^, Haiwei Gu^3^, Yan Jin^3^, Barry E. Bleske^4^, Matthew J. Campen^1^***

^1^ Department of Pharmaceutical Sciences, University of New Mexico College of Pharmacy, Albuquerque, NM USA 87131

^2^ University of New Mexico Prevention Research Center, University of New Mexico, Albuquerque, NM USA 87131

^3^ Center for Translational Sciences, Florida International University, Port St. Lucie, FL 34987

4 Department of Pharmacy Practice and Administrative Sciences, University of New Mexico, Albuquerque, NM USA 87131

*To whom correspondence should be addressed:

Matthew J Campen, PhD, Department of Pharmaceutical Sciences, MSC09 5360, 1 University of New Mexico, Albuquerque, NM 87131-0001, (505) 272-5569, [MCampen@salud.unm.edu](mailto:MCampen@salud.unm.edu)

**Supplemental table 1:** Lipid Ontology table of terms from students t-test of Standard Chow diet filtered air group versus Standard Chow diet ozone exposed group.

| Term ID | Description | Annotated | p-value | FDR q-value | -LOG(FDR-qvalue) |
| --- | --- | --- | --- | --- | --- |
| LION:0000070 | 1-alkyl,2-acylglycerophosphates [GP1002] | 4 | 0.00768 | 3.88E-02 | 1.411168 |
| LION:0000031 | 1-alkyl,2-acylglycerophosphocholines [GP0102] | 24 | 0.57156 | 7.60E-01 | 0.119186 |
| LION:0000039 | 1-alkyl,2-acylglycerophosphoethanolamines [GP0202] | 8 | 0.59855 | 7.85E-01 | 0.10513 |
| LION:0000060 | 1-alkyl,2-acylglycerophosphoglycerols [GP0402] | 5 | 0.08678 | 2.14E-01 | 0.669586 |
| LION:0000054 | 1-alkyl,2-acylglycerophosphoserines [GP0302] | 16 | 0.00036 | 4.38E-03 | 2.358526 |
| LION:0080974 | above average bilayer thickness | 33 | 0.18089 | 3.97E-01 | 0.401209 |
| LION:0080982 | above average lateral diffusion | 49 | 5.90E-06 | 1.99E-04 | 3.701147 |
| LION:0001740 | above average transition temperature | 38 | 0.03661 | 1.28E-01 | 0.89279 |
| LION:0080970 | average bilayer thickness | 30 | 0.14251 | 3.27E-01 | 0.485452 |
| LION:0080978 | average lateral diffusion | 25 | 0.45145 | 7.12E-01 | 0.14752 |
| LION:0001737 | average transition temperature | 27 | 0.03096 | 1.12E-01 | 0.950782 |
| LION:0080973 | below average bilayer thickness | 44 | 0.0002 | 2.89E-03 | 2.539102 |
| LION:0080981 | below average lateral diffusion | 35 | 0.30927 | 5.71E-01 | 0.243364 |
| LION:0001741 | below average transition temperature | 52 | 0.00359 | 2.13E-02 | 1.67162 |
| LION:0000254 | C12:0 | 3 | 0.08197 | 2.07E-01 | 0.68403 |
| LION:0000259 | C14:0 | 3 | 0.35609 | 5.99E-01 | 0.222573 |
| LION:0022229 | C15:0 | 3 | 0.90623 | 9.63E-01 | 0.016374 |
| LION:0002882 | C16:0 | 3 | 0.53325 | 7.48E-01 | 0.126098 |
| LION:0002900 | C16:1 | 3 | 0.50484 | 7.38E-01 | 0.131944 |
| LION:0022231 | C17:0 | 5 | 0.04526 | 1.43E-01 | 0.844664 |
| LION:0002921 | C18:0 | 3 | 0.54992 | 7.58E-01 | 0.120331 |
| LION:0002923 | C18:2 | 3 | 0.65644 | 8.19E-01 | 0.086716 |
| LION:0002924 | C18:3 | 4 | 0.30058 | 5.71E-01 | 0.243364 |
| LION:0002930 | C20:5 | 3 | 0.02624 | 9.82E-02 | 1.007889 |
| LION:0012446 | Ceramide 1-phosphates [SP0205] | 81 | 0.56311 | 7.58E-01 | 0.120331 |
| LION:0000084 | ceramide phosphocholines (sphingomyelins) [SP0301] | 11 | 0.04084 | 1.33E-01 | 0.876148 |
| LION:0000077 | ceramides [SP02] | 152 | 0.60646 | 7.85E-01 | 0.10513 |
| LION:0000467 | contains ether-bond | 57 | 0.00122 | 1.03E-02 | 1.987163 |
| LION:0000030 | diacylglycerophosphocholines [GP0101] | 43 | 0.07473 | 1.95E-01 | 0.709965 |
| LION:0000038 | diacylglycerophosphoethanolamines [GP0201] | 19 | 0.27514 | 5.45E-01 | 0.263603 |
| LION:0000059 | diacylglycerophosphoglycerols [GP0401] | 25 | 0.00159 | 1.24E-02 | 1.906578 |
| LION:0000047 | diacylglycerophosphoinositols [GP0601] | 12 | 0.00092 | 8.45E-03 | 2.073143 |
| LION:0000053 | diacylglycerophosphoserines [GP0301] | 24 | 0.09859 | 2.37E-01 | 0.625252 |
| LION:0012080 | endoplasmic reticulum (ER) | 272 | 0.1912 | 4.11E-01 | 0.386158 |
| LION:0012086 | endosome/lysosome | 13 | 0.19801 | 4.17E-01 | 0.379864 |
| LION:0002947 | fatty acid with 13-15 carbons | 8 | 0.86052 | 9.25E-01 | 0.033858 |
| LION:0002953 | fatty acid with 14 carbons | 5 | 0.68209 | 8.20E-01 | 0.086186 |
| LION:0002955 | fatty acid with 16 carbons | 6 | 0.31953 | 5.74E-01 | 0.241088 |
| LION:0002948 | fatty acid with 16-18 carbons | 23 | 0.15589 | 3.50E-01 | 0.455932 |
| LION:0002957 | fatty acid with 18 carbons | 12 | 0.48823 | 7.38E-01 | 0.131944 |
| LION:0000100 | fatty acid with 18 carbons or less | 34 | 0.70039 | 8.21E-01 | 0.085657 |
| LION:0002949 | fatty acid with 19-21 carbons | 17 | 0.46569 | 7.24E-01 | 0.140261 |
| LION:0002970 | fatty acid with 2 double bonds | 6 | 0.91765 | 9.65E-01 | 0.015473 |
| LION:0002959 | fatty acid with 20 carbons | 11 | 0.31075 | 5.71E-01 | 0.243364 |
| LION:0002961 | fatty acid with 22 carbons | 7 | 0.35206 | 5.99E-01 | 0.222573 |
| LION:0002950 | fatty acid with 22-24 carbons | 12 | 0.5009 | 7.38E-01 | 0.131944 |
| LION:0002963 | fatty acid with 24 carbons | 4 | 0.76543 | 8.69E-01 | 0.06098 |
| LION:0002965 | fatty acid with 26 carbons | 3 | 0.36588 | 6.06E-01 | 0.217527 |
| LION:0002971 | fatty acid with 3 double bonds | 9 | 0.40863 | 6.66E-01 | 0.176526 |
| LION:0002977 | fatty acid with 3-5 double bonds | 18 | 0.0654 | 1.83E-01 | 0.737549 |
| LION:0002972 | fatty acid with 4 double bonds | 4 | 0.82577 | 9.25E-01 | 0.033858 |
| LION:0002973 | fatty acid with 5 double bonds | 5 | 0.00232 | 1.56E-02 | 1.806875 |
| LION:0002966 | fatty acid with less than 2 double bonds | 42 | 0.51142 | 7.38E-01 | 0.131944 |
| LION:0002945 | fatty acid with more than 18 carbons | 34 | 0.32372 | 5.74E-01 | 0.241088 |
| LION:0002951 | fatty acid with more than 24 carbons | 5 | 0.05229 | 1.55E-01 | 0.809668 |
| LION:0002976 | fatty acid with more than 3 double bonds | 20 | 0.02139 | 8.64E-02 | 1.063486 |
| LION:0000001 | fatty acids [FA] | 24 | 0.84698 | 9.25E-01 | 0.033858 |
| LION:0001742 | fatty acids and conjugates [FA01] | 24 | 0.84698 | 9.25E-01 | 0.033858 |
| LION:0002958 | fatty acids with 19 carbons | 4 | 0.85162 | 9.25E-01 | 0.033858 |
| LION:0000002 | glycerolipids [GL] | 20 | 1 | 1.00E+00 | 0 |
| LION:0000019 | glycerophosphates [GP10] | 5 | 0.05229 | 1.55E-01 | 0.809668 |
| LION:0000010 | glycerophosphocholines [GP01] | 77 | 0.07148 | 1.95E-01 | 0.709965 |
| LION:0000011 | glycerophosphoethanolamines [GP02] | 30 | 0.30466 | 5.71E-01 | 0.243364 |
| LION:0000014 | glycerophosphoglycerols [GP04] | 31 | 0.00039 | 4.38E-03 | 2.358526 |
| LION:0000012 | glycerophosphoinositols [GP06] | 13 | 0.00457 | 2.56E-02 | 1.59176 |
| LION:0000003 | glycerophospholipids [GP] | 202 | 4.30E-09 | 2.17E-07 | 6.66354 |
| LION:0000013 | glycerophosphoserines [GP03] | 42 | 0.00197 | 1.42E-02 | 1.847712 |
| LION:0012085 | golgi apparatus | 12 | 0.02277 | 8.85E-02 | 1.053057 |
| LION:0000093 | headgroup with negative charge | 117 | 2.00E-05 | 5.05E-04 | 3.296709 |
| LION:0000094 | headgroup with neutral charge | 20 | 1 | 1.00E+00 | 0 |
| LION:0000095 | headgroup with positive charge / zwitter-ion | 118 | 0.01805 | 7.60E-02 | 1.119186 |
| LION:0080971 | high bilayer thickness | 17 | 0.33684 | 5.87E-01 | 0.231362 |
| LION:0080979 | high lateral diffusion | 30 | 0.00616 | 3.27E-02 | 1.485452 |
| LION:0001738 | high transition temperature | 19 | 0.55834 | 7.58E-01 | 0.120331 |
| LION:0012084 | lipid droplet | 19 | 1 | 1.00E+00 | 0 |
| LION:0012011 | lipid storage | 19 | 1 | 1.00E+00 | 0 |
| LION:0012009 | lipid-mediated signalling | 177 | 0.61681 | 7.89E-01 | 0.102923 |
| LION:0080969 | low bilayer thickness | 29 | 0.03943 | 1.33E-01 | 0.876148 |
| LION:0080977 | low lateral diffusion | 17 | 0.67946 | 8.20E-01 | 0.086186 |
| LION:0001736 | low transition temperature | 34 | 0.05767 | 1.66E-01 | 0.779892 |
| LION:0000599 | lysoglycerophospholipids | 16 | 0.70173 | 8.21E-01 | 0.085657 |
| LION:0012010 | membrane component | 212 | 1.30E-10 | 1.31E-08 | 7.882729 |
| LION:0012081 | mitochondrion | 63 | 0.00865 | 4.16E-02 | 1.380907 |
| LION:0000034 | monoacylglycerophosphocholines [GP0105] | 9 | 0.26425 | 5.45E-01 | 0.263603 |
| LION:0000042 | monoacylglycerophosphoethanolamines [GP0205] | 3 | 0.67326 | 8.20E-01 | 0.086186 |
| LION:0002969 | monounsaturated fatty acid | 15 | 0.7151 | 8.21E-01 | 0.085657 |
| LION:0012441 | N-acylsphingosines (ceramides) [SP0201] | 71 | 0.7109 | 8.21E-01 | 0.085657 |
| LION:0000464 | negative intrinsic curvature | 187 | 0.50281 | 7.38E-01 | 0.131944 |
| LION:0000465 | neutral intrinsic curvature | 107 | 5.00E-05 | 1.01E-03 | 2.995679 |
| LION:0012082 | plasma membrane | 207 | 0.42766 | 6.86E-01 | 0.163676 |
| LION:0002967 | polyunsaturated fatty acid | 26 | 0.07542 | 1.95E-01 | 0.709965 |
| LION:0000466 | positive intrinsic curvature | 45 | 0.00267 | 1.69E-02 | 1.772113 |
| LION:0002968 | saturated fatty acid | 27 | 0.62613 | 7.90E-01 | 0.102373 |
| LION:0000004 | sphingolipids [SP] | 164 | 0.53086 | 7.48E-01 | 0.126098 |
| LION:0000622 | triacylglycerols [GL0301] | 18 | 1 | 1.00E+00 | 0 |
| LION:0080972 | very high bilayer thickness | 16 | 0.27491 | 5.45E-01 | 0.263603 |
| LION:0080980 | very high lateral diffusion | 21 | 0.00013 | 2.19E-03 | 2.659556 |
| LION:0001739 | very high transition temperature | 19 | 0.00952 | 4.37E-02 | 1.359519 |
| LION:0080968 | very low bilayer thickness | 15 | 0.00047 | 4.75E-03 | 2.323306 |
| LION:0080976 | very low lateral diffusion | 19 | 0.14197 | 3.27E-01 | 0.485452 |
| LION:0001735 | very low transition temperature | 18 | 0.01442 | 6.33E-02 | 1.198596 |

**Supplemental table 2:** Lipid Ontology table of terms from students t-test of Soybean Oil diet filtered air group versus Soybean Oil diet ozone exposed group.

| Term ID | Description | Annotated | p-value | FDR q-value | -LOG (FDR q-value) |
| --- | --- | --- | --- | --- | --- |
| LION:0000070 | 1-alkyl,2-acylglycerophosphates [GP1002] | 4 | 0.99761 | 9.98E-01 | 0.000869 |
| LION:0000031 | 1-alkyl,2-acylglycerophosphocholines [GP0102] | 24 | 0.34325 | 4.95E-01 | 0.305395 |
| LION:0000039 | 1-alkyl,2-acylglycerophosphoethanolamines [GP0202] | 8 | 0.14172 | 2.35E-01 | 0.628932 |
| LION:0000060 | 1-alkyl,2-acylglycerophosphoglycerols [GP0402] | 5 | 0.00418 | 1.92E-02 | 1.716699 |
| LION:0000054 | 1-alkyl,2-acylglycerophosphoserines [GP0302] | 16 | 0.04041 | 1.02E-01 | 0.9914 |
| LION:0080974 | above average bilayer thickness | 33 | 0.011 | 3.98E-02 | 1.400117 |
| LION:0080982 | above average lateral diffusion | 49 | 1.30E-08 | 4.38E-07 | 6.358526 |
| LION:0001740 | above average transition temperature | 38 | 0.03556 | 9.21E-02 | 1.03574 |
| LION:0080970 | average bilayer thickness | 30 | 0.1931 | 3.02E-01 | 0.519993 |
| LION:0080978 | average lateral diffusion | 25 | 0.56586 | 7.14E-01 | 0.146302 |
| LION:0001737 | average transition temperature | 27 | 0.11463 | 2.06E-01 | 0.686133 |
| LION:0080973 | below average bilayer thickness | 44 | 0.00033 | 3.03E-03 | 2.518557 |
| LION:0080981 | below average lateral diffusion | 35 | 0.19429 | 3.02E-01 | 0.519993 |
| LION:0001741 | below average transition temperature | 52 | 1.80E-05 | 2.27E-04 | 3.643974 |
| LION:0000254 | C12:0 | 3 | 0.11614 | 2.06E-01 | 0.686133 |
| LION:0000259 | C14:0 | 3 | 0.042 | 1.03E-01 | 0.987163 |
| LION:0022229 | C15:0 | 3 | 0.95417 | 9.98E-01 | 0.000869 |
| LION:0002882 | C16:0 | 3 | 0.06767 | 1.52E-01 | 0.818156 |
| LION:0002900 | C16:1 | 3 | 0.5309 | 6.79E-01 | 0.16813 |
| LION:0022231 | C17:0 | 5 | 0.13735 | 2.31E-01 | 0.636388 |
| LION:0002921 | C18:0 | 3 | 0.0813 | 1.64E-01 | 0.785156 |
| LION:0002923 | C18:2 | 3 | 0.5999 | 7.39E-01 | 0.131356 |
| LION:0002924 | C18:3 | 4 | 0.90965 | 9.98E-01 | 0.000869 |
| LION:0002930 | C20:5 | 3 | 0.00174 | 1.10E-02 | 1.958607 |
| LION:0012446 | Ceramide 1-phosphates [SP0205] | 81 | 0.43829 | 5.87E-01 | 0.231362 |
| LION:0000084 | ceramide phosphocholines (sphingomyelins) [SP0301] | 11 | 0.07365 | 1.55E-01 | 0.809668 |
| LION:0000077 | ceramides [SP02] | 152 | 0.7695 | 9.04E-01 | 0.043832 |
| LION:0000467 | contains ether-bond | 57 | 0.07637 | 1.57E-01 | 0.8041 |
| LION:0000030 | diacylglycerophosphocholines [GP0101] | 43 | 0.41421 | 5.81E-01 | 0.235824 |
| LION:0000038 | diacylglycerophosphoethanolamines [GP0201] | 19 | 0.00414 | 1.92E-02 | 1.716699 |
| LION:0000059 | diacylglycerophosphoglycerols [GP0401] | 25 | 0.00071 | 5.98E-03 | 2.223299 |
| LION:0000047 | diacylglycerophosphoinositols [GP0601] | 12 | 0.01703 | 5.38E-02 | 1.269218 |
| LION:0000053 | diacylglycerophosphoserines [GP0301] | 24 | 0.16177 | 2.64E-01 | 0.578396 |
| LION:0012080 | endoplasmic reticulum (ER) | 272 | 0.3803 | 5.41E-01 | 0.266803 |
| LION:0012086 | endosome/lysosome | 13 | 0.10805 | 2.02E-01 | 0.694649 |
| LION:0002947 | fatty acid with 13-15 carbons | 8 | 0.5756 | 7.18E-01 | 0.143876 |
| LION:0002953 | fatty acid with 14 carbons | 5 | 0.09341 | 1.78E-01 | 0.74958 |
| LION:0002955 | fatty acid with 16 carbons | 6 | 0.06983 | 1.53E-01 | 0.815309 |
| LION:0002948 | fatty acid with 16-18 carbons | 23 | 0.01659 | 5.38E-02 | 1.269218 |
| LION:0002957 | fatty acid with 18 carbons | 12 | 0.32067 | 4.76E-01 | 0.322393 |
| LION:0000100 | fatty acid with 18 carbons or less | 34 | 0.01098 | 3.98E-02 | 1.400117 |
| LION:0002949 | fatty acid with 19-21 carbons | 17 | 0.08593 | 1.70E-01 | 0.769551 |
| LION:0002970 | fatty acid with 2 double bonds | 6 | 0.86692 | 9.98E-01 | 0.000869 |
| LION:0002959 | fatty acid with 20 carbons | 11 | 0.02171 | 6.64E-02 | 1.177832 |
| LION:0002961 | fatty acid with 22 carbons | 7 | 0.05991 | 1.38E-01 | 0.860121 |
| LION:0002950 | fatty acid with 22-24 carbons | 12 | 0.44285 | 5.87E-01 | 0.231362 |
| LION:0002963 | fatty acid with 24 carbons | 4 | 0.94866 | 9.98E-01 | 0.000869 |
| LION:0002965 | fatty acid with 26 carbons | 3 | 0.4478 | 5.87E-01 | 0.231362 |
| LION:0002971 | fatty acid with 3 double bonds | 9 | 0.91328 | 9.98E-01 | 0.000869 |
| LION:0002977 | fatty acid with 3-5 double bonds | 18 | 0.01103 | 3.98E-02 | 1.400117 |
| LION:0002972 | fatty acid with 4 double bonds | 4 | 0.00228 | 1.28E-02 | 1.89279 |
| LION:0002973 | fatty acid with 5 double bonds | 5 | 2.30E-05 | 2.58E-04 | 3.58838 |
| LION:0002966 | fatty acid with less than 2 double bonds | 42 | 0.02817 | 8.05E-02 | 1.094204 |
| LION:0002945 | fatty acid with more than 18 carbons | 34 | 0.11987 | 2.09E-01 | 0.679854 |
| LION:0002951 | fatty acid with more than 24 carbons | 5 | 0.24671 | 3.72E-01 | 0.429457 |
| LION:0002976 | fatty acid with more than 3 double bonds | 20 | 0.00159 | 1.07E-02 | 1.970616 |
| LION:0000001 | fatty acids [FA] | 24 | 0.04642 | 1.09E-01 | 0.962574 |
| LION:0001742 | fatty acids and conjugates [FA01] | 24 | 0.04642 | 1.09E-01 | 0.962574 |
| LION:0002958 | fatty acids with 19 carbons | 4 | 0.89112 | 9.98E-01 | 0.000869 |
| LION:0000002 | glycerolipids [GL] | 20 | 0.99159 | 9.98E-01 | 0.000869 |
| LION:0000019 | glycerophosphates [GP10] | 5 | 0.68209 | 8.20E-01 | 0.086186 |
| LION:0000010 | glycerophosphocholines [GP01] | 77 | 0.34192 | 4.95E-01 | 0.305395 |
| LION:0000011 | glycerophosphoethanolamines [GP02] | 30 | 0.00227 | 1.28E-02 | 1.89279 |
| LION:0000014 | glycerophosphoglycerols [GP04] | 31 | 5.60E-06 | 8.08E-05 | 4.092589 |
| LION:0000012 | glycerophosphoinositols [GP06] | 13 | 0.00903 | 3.80E-02 | 1.420216 |
| LION:0000003 | glycerophospholipids [GP] | 202 | 6.70E-10 | 4.75E-08 | 7.323306 |
| LION:0000013 | glycerophosphoserines [GP03] | 42 | 0.02977 | 8.13E-02 | 1.089909 |
| LION:0012085 | golgi apparatus | 12 | 0.1668 | 2.67E-01 | 0.573489 |
| LION:0000093 | headgroup with negative charge | 117 | 2.50E-07 | 5.05E-06 | 5.296709 |
| LION:0000094 | headgroup with neutral charge | 20 | 0.99159 | 9.98E-01 | 0.000869 |
| LION:0000095 | headgroup with positive charge / zwitter-ion | 118 | 0.0046 | 2.02E-02 | 1.694649 |
| LION:0080971 | high bilayer thickness | 17 | 0.07163 | 1.54E-01 | 0.812479 |
| LION:0080979 | high lateral diffusion | 30 | 0.00107 | 7.72E-03 | 2.112383 |
| LION:0001738 | high transition temperature | 19 | 0.5147 | 6.66E-01 | 0.176526 |
| LION:0012084 | lipid droplet | 19 | 0.99163 | 9.98E-01 | 0.000869 |
| LION:0012011 | lipid storage | 19 | 0.99163 | 9.98E-01 | 0.000869 |
| LION:0012009 | lipid-mediated signalling | 177 | 0.98915 | 9.98E-01 | 0.000869 |
| LION:0080969 | low bilayer thickness | 29 | 0.01645 | 5.38E-02 | 1.269218 |
| LION:0080977 | low lateral diffusion | 17 | 0.43175 | 5.87E-01 | 0.231362 |
| LION:0001736 | low transition temperature | 34 | 0.00369 | 1.92E-02 | 1.716699 |
| LION:0000599 | lysoglycerophospholipids | 16 | 0.02871 | 8.05E-02 | 1.094204 |
| LION:0012010 | membrane component | 212 | 9.40E-10 | 4.75E-08 | 7.323306 |
| LION:0012081 | mitochondrion | 63 | 2.30E-07 | 5.05E-06 | 5.296709 |
| LION:0000034 | monoacylglycerophosphocholines [GP0105] | 9 | 0.09073 | 1.76E-01 | 0.754487 |
| LION:0000042 | monoacylglycerophosphoethanolamines [GP0205] | 3 | 0.22571 | 3.45E-01 | 0.462181 |
| LION:0002969 | monounsaturated fatty acid | 15 | 0.44163 | 5.87E-01 | 0.231362 |
| LION:0012441 | N-acylsphingosines (ceramides) [SP0201] | 71 | 0.89208 | 9.98E-01 | 0.000869 |
| LION:0000464 | negative intrinsic curvature | 187 | 0.96326 | 9.98E-01 | 0.000869 |
| LION:0000465 | neutral intrinsic curvature | 107 | 0.02732 | 8.05E-02 | 1.094204 |
| LION:0012082 | plasma membrane | 207 | 0.68193 | 8.20E-01 | 0.086186 |
| LION:0002967 | polyunsaturated fatty acid | 26 | 0.01349 | 4.70E-02 | 1.327902 |
| LION:0000466 | positive intrinsic curvature | 45 | 0.00079 | 6.14E-03 | 2.211832 |
| LION:0002968 | saturated fatty acid | 27 | 0.01022 | 3.98E-02 | 1.400117 |
| LION:0000004 | sphingolipids [SP] | 164 | 0.74401 | 8.84E-01 | 0.053548 |
| LION:0000622 | triacylglycerols [GL0301] | 18 | 0.9917 | 9.98E-01 | 0.000869 |
| LION:0080972 | very high bilayer thickness | 16 | 0.12379 | 2.12E-01 | 0.673664 |
| LION:0080980 | very high lateral diffusion | 21 | 3.00E-07 | 5.05E-06 | 5.296709 |
| LION:0001739 | very high transition temperature | 19 | 0.03431 | 9.12E-02 | 1.040005 |
| LION:0080968 | very low bilayer thickness | 15 | 0.00011 | 1.11E-03 | 2.954677 |
| LION:0080976 | very low lateral diffusion | 19 | 0.11079 | 2.03E-01 | 0.692504 |
| LION:0001735 | very low transition temperature | 18 | 0.0038 | 1.92E-02 | 1.716699 |

**Supplemental table 3**: Lipid Ontology table of terms from students t-test of Flaxseed Oil diet filtered air group versus Flaxseed Oil diet ozone-exposed group.

| Term ID | Description | Annotated | p-value | FDR q-value | -LOG (FDR q-value) |
| --- | --- | --- | --- | --- | --- |
| LION:0000070 | 1-alkyl,2-acylglycerophosphates [GP1002] | 4 | 0.98517 | 9.96E-01 | 0.001741 |
| LION:0000031 | 1-alkyl,2-acylglycerophosphocholines [GP0102] | 24 | 0.69149 | 8.12E-01 | 0.090444 |
| LION:0000039 | 1-alkyl,2-acylglycerophosphoethanolamines [GP0202] | 8 | 0.78291 | 8.88E-01 | 0.051587 |
| LION:0000060 | 1-alkyl,2-acylglycerophosphoglycerols [GP0402] | 5 | 0.05229 | 1.32E-01 | 0.879426 |
| LION:0000054 | 1-alkyl,2-acylglycerophosphoserines [GP0302] | 16 | 0.18156 | 3.00E-01 | 0.522879 |
| LION:0080974 | above average bilayer thickness | 33 | 0.37009 | 5.21E-01 | 0.283162 |
| LION:0080982 | above average lateral diffusion | 49 | 1.10E-05 | 1.85E-04 | 3.732828 |
| LION:0001740 | above average transition temperature | 38 | 0.42599 | 5.81E-01 | 0.235824 |
| LION:0080970 | average bilayer thickness | 30 | 0.18692 | 3.00E-01 | 0.522879 |
| LION:0080978 | average lateral diffusion | 25 | 0.58052 | 7.36E-01 | 0.133122 |
| LION:0001737 | average transition temperature | 27 | 0.00912 | 3.29E-02 | 1.482804 |
| LION:0080973 | below average bilayer thickness | 44 | 4.90E-05 | 5.61E-04 | 3.251037 |
| LION:0080981 | below average lateral diffusion | 35 | 0.60617 | 7.47E-01 | 0.126679 |
| LION:0001741 | below average transition temperature | 52 | 0.00185 | 1.04E-02 | 1.982967 |
| LION:0000254 | C12:0 | 3 | 0.75024 | 8.61E-01 | 0.064997 |
| LION:0000259 | C14:0 | 3 | 0.59064 | 7.36E-01 | 0.133122 |
| LION:0022229 | C15:0 | 3 | 0.83851 | 9.41E-01 | 0.02641 |
| LION:0002882 | C16:0 | 3 | 0.06456 | 1.45E-01 | 0.838632 |
| LION:0002900 | C16:1 | 3 | 0.4061 | 5.62E-01 | 0.250264 |
| LION:0022231 | C17:0 | 5 | 0.49034 | 6.52E-01 | 0.185752 |
| LION:0002921 | C18:0 | 3 | 0.01392 | 4.69E-02 | 1.328827 |
| LION:0002923 | C18:2 | 3 | 0.04725 | 1.22E-01 | 0.91364 |
| LION:0002924 | C18:3 | 4 | 0.18462 | 3.00E-01 | 0.522879 |
| LION:0002930 | C20:5 | 3 | 0.03017 | 8.96E-02 | 1.047692 |
| LION:0012446 | Ceramide 1-phosphates [SP0205] | 81 | 0.71206 | 8.27E-01 | 0.082494 |
| LION:0000084 | ceramide phosphocholines (sphingomyelins) [SP0301] | 11 | 0.16254 | 2.78E-01 | 0.555955 |
| LION:0000077 | ceramides [SP02] | 152 | 0.98255 | 9.96E-01 | 0.001741 |
| LION:0000467 | contains ether-bond | 57 | 0.64017 | 7.79E-01 | 0.108463 |
| LION:0000030 | diacylglycerophosphocholines [GP0101] | 43 | 0.04188 | 1.14E-01 | 0.943095 |
| LION:0000038 | diacylglycerophosphoethanolamines [GP0201] | 19 | 0.10124 | 1.97E-01 | 0.705534 |
| LION:0000059 | diacylglycerophosphoglycerols [GP0401] | 25 | 7.80E-06 | 1.58E-04 | 3.801343 |
| LION:0000047 | diacylglycerophosphoinositols [GP0601] | 12 | 0.0634 | 1.45E-01 | 0.838632 |
| LION:0000053 | diacylglycerophosphoserines [GP0301] | 24 | 0.11314 | 2.08E-01 | 0.681937 |
| LION:0012080 | endoplasmic reticulum (ER) | 272 | 0.89927 | 9.96E-01 | 0.001741 |
| LION:0012086 | endosome/lysosome | 13 | 0.58786 | 7.36E-01 | 0.133122 |
| LION:0002947 | fatty acid with 13-15 carbons | 8 | 0.31888 | 4.86E-01 | 0.313364 |
| LION:0002953 | fatty acid with 14 carbons | 5 | 0.16529 | 2.78E-01 | 0.555955 |
| LION:0002955 | fatty acid with 16 carbons | 6 | 0.06591 | 1.45E-01 | 0.838632 |
| LION:0002948 | fatty acid with 16-18 carbons | 23 | 5.00E-05 | 5.61E-04 | 3.251037 |
| LION:0002957 | fatty acid with 18 carbons | 12 | 0.00044 | 4.04E-03 | 2.393619 |
| LION:0000100 | fatty acid with 18 carbons or less | 34 | 0.00151 | 1.02E-02 | 1.9914 |
| LION:0002949 | fatty acid with 19-21 carbons | 17 | 0.08216 | 1.69E-01 | 0.772113 |
| LION:0002970 | fatty acid with 2 double bonds | 6 | 0.10935 | 2.08E-01 | 0.681937 |
| LION:0002959 | fatty acid with 20 carbons | 11 | 0.14468 | 2.55E-01 | 0.59346 |
| LION:0002961 | fatty acid with 22 carbons | 7 | 0.06266 | 1.45E-01 | 0.838632 |
| LION:0002950 | fatty acid with 22-24 carbons | 12 | 0.32319 | 4.86E-01 | 0.313364 |
| LION:0002963 | fatty acid with 24 carbons | 4 | 0.92402 | 9.96E-01 | 0.001741 |
| LION:0002965 | fatty acid with 26 carbons | 3 | 0.95097 | 9.96E-01 | 0.001741 |
| LION:0002971 | fatty acid with 3 double bonds | 9 | 0.0973 | 1.93E-01 | 0.714443 |
| LION:0002977 | fatty acid with 3-5 double bonds | 18 | 0.00196 | 1.04E-02 | 1.982967 |
| LION:0002972 | fatty acid with 4 double bonds | 4 | 0.05672 | 1.40E-01 | 0.853872 |
| LION:0002973 | fatty acid with 5 double bonds | 5 | 0.06015 | 1.45E-01 | 0.838632 |
| LION:0002966 | fatty acid with less than 2 double bonds | 42 | 0.0929 | 1.88E-01 | 0.725842 |
| LION:0002945 | fatty acid with more than 18 carbons | 34 | 0.06823 | 1.47E-01 | 0.832683 |
| LION:0002951 | fatty acid with more than 24 carbons | 5 | 0.54871 | 7.11E-01 | 0.14813 |
| LION:0002976 | fatty acid with more than 3 double bonds | 20 | 0.00034 | 3.43E-03 | 2.464706 |
| LION:0000001 | fatty acids [FA] | 24 | 0.01922 | 6.07E-02 | 1.216811 |
| LION:0001742 | fatty acids and conjugates [FA01] | 24 | 0.01922 | 6.07E-02 | 1.216811 |
| LION:0002958 | fatty acids with 19 carbons | 4 | 0.51258 | 6.72E-01 | 0.172631 |
| LION:0000002 | glycerolipids [GL] | 20 | 0.00496 | 2.00E-02 | 1.69897 |
| LION:0000019 | glycerophosphates [GP10] | 5 | 0.98506 | 9.96E-01 | 0.001741 |
| LION:0000010 | glycerophosphocholines [GP01] | 77 | 0.14618 | 2.55E-01 | 0.59346 |
| LION:0000011 | glycerophosphoethanolamines [GP02] | 30 | 0.35041 | 5.06E-01 | 0.295849 |
| LION:0000014 | glycerophosphoglycerols [GP04] | 31 | 1.90E-06 | 6.40E-05 | 4.19382 |
| LION:0000012 | glycerophosphoinositols [GP06] | 13 | 0.03518 | 9.87E-02 | 1.005683 |
| LION:0000003 | glycerophospholipids [GP] | 202 | 2.20E-05 | 3.17E-04 | 3.498941 |
| LION:0000013 | glycerophosphoserines [GP03] | 42 | 0.08129 | 1.69E-01 | 0.772113 |
| LION:0012085 | golgi apparatus | 12 | 0.35035 | 5.06E-01 | 0.295849 |
| LION:0000093 | headgroup with negative charge | 117 | 8.90E-07 | 4.49E-05 | 4.347754 |
| LION:0000094 | headgroup with neutral charge | 20 | 0.00496 | 2.00E-02 | 1.69897 |
| LION:0000095 | headgroup with positive charge / zwitter-ion | 118 | 0.02859 | 8.75E-02 | 1.057992 |
| LION:0080971 | high bilayer thickness | 17 | 0.32707 | 4.86E-01 | 0.313364 |
| LION:0080979 | high lateral diffusion | 30 | 0.0113 | 3.94E-02 | 1.404504 |
| LION:0001738 | high transition temperature | 19 | 0.66142 | 7.86E-01 | 0.104577 |
| LION:0012084 | lipid droplet | 19 | 0.00311 | 1.37E-02 | 1.863279 |
| LION:0012011 | lipid storage | 19 | 0.00311 | 1.37E-02 | 1.863279 |
| LION:0012009 | lipid-mediated signalling | 177 | 0.98915 | 9.96E-01 | 0.001741 |
| LION:0080969 | low bilayer thickness | 29 | 0.00792 | 3.08E-02 | 1.511449 |
| LION:0080977 | low lateral diffusion | 17 | 0.6553 | 7.86E-01 | 0.104577 |
| LION:0001736 | low transition temperature | 34 | 0.00884 | 3.29E-02 | 1.482804 |
| LION:0000599 | lysoglycerophospholipids | 16 | 0.00091 | 7.07E-03 | 2.150581 |
| LION:0012010 | membrane component | 212 | 6.00E-06 | 1.51E-04 | 3.821023 |
| LION:0012081 | mitochondrion | 63 | 0.00249 | 1.26E-02 | 1.899629 |
| LION:0000034 | monoacylglycerophosphocholines [GP0105] | 9 | 0.03468 | 9.87E-02 | 1.005683 |
| LION:0000042 | monoacylglycerophosphoethanolamines [GP0205] | 3 | 0.04725 | 1.22E-01 | 0.91364 |
| LION:0002969 | monounsaturated fatty acid | 15 | 0.11096 | 2.08E-01 | 0.681937 |
| LION:0012441 | N-acylsphingosines (ceramides) [SP0201] | 71 | 0.99623 | 9.96E-01 | 0.001741 |
| LION:0000464 | negative intrinsic curvature | 187 | 0.98532 | 9.96E-01 | 0.001741 |
| LION:0000465 | neutral intrinsic curvature | 107 | 0.1271 | 2.29E-01 | 0.640165 |
| LION:0012082 | plasma membrane | 207 | 0.95638 | 9.96E-01 | 0.001741 |
| LION:0002967 | polyunsaturated fatty acid | 26 | 0.00049 | 4.12E-03 | 2.385103 |
| LION:0000466 | positive intrinsic curvature | 45 | 0.00135 | 9.74E-03 | 2.011441 |
| LION:0002968 | saturated fatty acid | 27 | 0.20785 | 3.28E-01 | 0.484126 |
| LION:0000004 | sphingolipids [SP] | 164 | 0.98601 | 9.96E-01 | 0.001741 |
| LION:0000622 | triacylglycerols [GL0301] | 18 | 0.00187 | 1.04E-02 | 1.982967 |
| LION:0080972 | very high bilayer thickness | 16 | 0.47231 | 6.36E-01 | 0.196543 |
| LION:0080980 | very high lateral diffusion | 21 | 8.30E-07 | 4.49E-05 | 4.347754 |
| LION:0001739 | very high transition temperature | 19 | 0.37141 | 5.21E-01 | 0.283162 |
| LION:0080968 | very low bilayer thickness | 15 | 0.00163 | 1.03E-02 | 1.987163 |
| LION:0080976 | very low lateral diffusion | 19 | 0.24332 | 3.78E-01 | 0.422508 |
| LION:0001735 | very low transition temperature | 18 | 0.00289 | 1.37E-02 | 1.863279 |

**Supplemental table 4:** Lipid Ontology table of terms from students t-test of Coconut Oil diet filtered air group versus Coconut Oil diet ozone-exposed group.

| Term ID | Description | Annotated | p-value | FDR q-value | -LOG (FDR q-value) |
| --- | --- | --- | --- | --- | --- |
| LION:0000070 | 1-alkyl,2-acylglycerophosphates [GP1002] | 4 | 0.00768 | 3.88E-02 | 1.411168 |
| LION:0000031 | 1-alkyl,2-acylglycerophosphocholines [GP0102] | 24 | 0.57156 | 7.60E-01 | 0.119186 |
| LION:0000039 | 1-alkyl,2-acylglycerophosphoethanolamines [GP0202] | 8 | 0.59855 | 7.85E-01 | 0.10513 |
| LION:0000060 | 1-alkyl,2-acylglycerophosphoglycerols [GP0402] | 5 | 0.08678 | 2.14E-01 | 0.669586 |
| LION:0000054 | 1-alkyl,2-acylglycerophosphoserines [GP0302] | 16 | 0.00036 | 4.38E-03 | 2.358526 |
| LION:0080974 | above average bilayer thickness | 33 | 0.18089 | 3.97E-01 | 0.401209 |
| LION:0080982 | above average lateral diffusion | 49 | 5.90E-06 | 1.99E-04 | 3.701147 |
| LION:0001740 | above average transition temperature | 38 | 0.03661 | 1.28E-01 | 0.89279 |
| LION:0080970 | average bilayer thickness | 30 | 0.14251 | 3.27E-01 | 0.485452 |
| LION:0080978 | average lateral diffusion | 25 | 0.45145 | 7.12E-01 | 0.14752 |
| LION:0001737 | average transition temperature | 27 | 0.03096 | 1.12E-01 | 0.950782 |
| LION:0080973 | below average bilayer thickness | 44 | 0.0002 | 2.89E-03 | 2.539102 |
| LION:0080981 | below average lateral diffusion | 35 | 0.30927 | 5.71E-01 | 0.243364 |
| LION:0001741 | below average transition temperature | 52 | 0.00359 | 2.13E-02 | 1.67162 |
| LION:0000254 | C12:0 | 3 | 0.08197 | 2.07E-01 | 0.68403 |
| LION:0000259 | C14:0 | 3 | 0.35609 | 5.99E-01 | 0.222573 |
| LION:0022229 | C15:0 | 3 | 0.90623 | 9.63E-01 | 0.016374 |
| LION:0002882 | C16:0 | 3 | 0.53325 | 7.48E-01 | 0.126098 |
| LION:0002900 | C16:1 | 3 | 0.50484 | 7.38E-01 | 0.131944 |
| LION:0022231 | C17:0 | 5 | 0.04526 | 1.43E-01 | 0.844664 |
| LION:0002921 | C18:0 | 3 | 0.54992 | 7.58E-01 | 0.120331 |
| LION:0002923 | C18:2 | 3 | 0.65644 | 8.19E-01 | 0.086716 |
| LION:0002924 | C18:3 | 4 | 0.30058 | 5.71E-01 | 0.243364 |
| LION:0002930 | C20:5 | 3 | 0.02624 | 9.82E-02 | 1.007889 |
| LION:0012446 | Ceramide 1-phosphates [SP0205] | 81 | 0.56311 | 7.58E-01 | 0.120331 |
| LION:0000084 | ceramide phosphocholines (sphingomyelins) [SP0301] | 11 | 0.04084 | 1.33E-01 | 0.876148 |
| LION:0000077 | ceramides [SP02] | 152 | 0.60646 | 7.85E-01 | 0.10513 |
| LION:0000467 | contains ether-bond | 57 | 0.00122 | 1.03E-02 | 1.987163 |
| LION:0000030 | diacylglycerophosphocholines [GP0101] | 43 | 0.07473 | 1.95E-01 | 0.709965 |
| LION:0000038 | diacylglycerophosphoethanolamines [GP0201] | 19 | 0.27514 | 5.45E-01 | 0.263603 |
| LION:0000059 | diacylglycerophosphoglycerols [GP0401] | 25 | 0.00159 | 1.24E-02 | 1.906578 |
| LION:0000047 | diacylglycerophosphoinositols [GP0601] | 12 | 0.00092 | 8.45E-03 | 2.073143 |
| LION:0000053 | diacylglycerophosphoserines [GP0301] | 24 | 0.09859 | 2.37E-01 | 0.625252 |
| LION:0012080 | endoplasmic reticulum (ER) | 272 | 0.1912 | 4.11E-01 | 0.386158 |
| LION:0012086 | endosome/lysosome | 13 | 0.19801 | 4.17E-01 | 0.379864 |
| LION:0002947 | fatty acid with 13-15 carbons | 8 | 0.86052 | 9.25E-01 | 0.033858 |
| LION:0002953 | fatty acid with 14 carbons | 5 | 0.68209 | 8.20E-01 | 0.086186 |
| LION:0002955 | fatty acid with 16 carbons | 6 | 0.31953 | 5.74E-01 | 0.241088 |
| LION:0002948 | fatty acid with 16-18 carbons | 23 | 0.15589 | 3.50E-01 | 0.455932 |
| LION:0002957 | fatty acid with 18 carbons | 12 | 0.48823 | 7.38E-01 | 0.131944 |
| LION:0000100 | fatty acid with 18 carbons or less | 34 | 0.70039 | 8.21E-01 | 0.085657 |
| LION:0002949 | fatty acid with 19-21 carbons | 17 | 0.46569 | 7.24E-01 | 0.140261 |
| LION:0002970 | fatty acid with 2 double bonds | 6 | 0.91765 | 9.65E-01 | 0.015473 |
| LION:0002959 | fatty acid with 20 carbons | 11 | 0.31075 | 5.71E-01 | 0.243364 |
| LION:0002961 | fatty acid with 22 carbons | 7 | 0.35206 | 5.99E-01 | 0.222573 |
| LION:0002950 | fatty acid with 22-24 carbons | 12 | 0.5009 | 7.38E-01 | 0.131944 |
| LION:0002963 | fatty acid with 24 carbons | 4 | 0.76543 | 8.69E-01 | 0.06098 |
| LION:0002965 | fatty acid with 26 carbons | 3 | 0.36588 | 6.06E-01 | 0.217527 |
| LION:0002971 | fatty acid with 3 double bonds | 9 | 0.40863 | 6.66E-01 | 0.176526 |
| LION:0002977 | fatty acid with 3-5 double bonds | 18 | 0.0654 | 1.83E-01 | 0.737549 |
| LION:0002972 | fatty acid with 4 double bonds | 4 | 0.82577 | 9.25E-01 | 0.033858 |
| LION:0002973 | fatty acid with 5 double bonds | 5 | 0.00232 | 1.56E-02 | 1.806875 |
| LION:0002966 | fatty acid with less than 2 double bonds | 42 | 0.51142 | 7.38E-01 | 0.131944 |
| LION:0002945 | fatty acid with more than 18 carbons | 34 | 0.32372 | 5.74E-01 | 0.241088 |
| LION:0002951 | fatty acid with more than 24 carbons | 5 | 0.05229 | 1.55E-01 | 0.809668 |
| LION:0002976 | fatty acid with more than 3 double bonds | 20 | 0.02139 | 8.64E-02 | 1.063486 |
| LION:0000001 | fatty acids [FA] | 24 | 0.84698 | 9.25E-01 | 0.033858 |
| LION:0001742 | fatty acids and conjugates [FA01] | 24 | 0.84698 | 9.25E-01 | 0.033858 |
| LION:0002958 | fatty acids with 19 carbons | 4 | 0.85162 | 9.25E-01 | 0.033858 |
| LION:0000002 | glycerolipids [GL] | 20 | 1 | 1.00E+00 | 0 |
| LION:0000019 | glycerophosphates [GP10] | 5 | 0.05229 | 1.55E-01 | 0.809668 |
| LION:0000010 | glycerophosphocholines [GP01] | 77 | 0.07148 | 1.95E-01 | 0.709965 |
| LION:0000011 | glycerophosphoethanolamines [GP02] | 30 | 0.30466 | 5.71E-01 | 0.243364 |
| LION:0000014 | glycerophosphoglycerols [GP04] | 31 | 0.00039 | 4.38E-03 | 2.358526 |
| LION:0000012 | glycerophosphoinositols [GP06] | 13 | 0.00457 | 2.56E-02 | 1.59176 |
| LION:0000003 | glycerophospholipids [GP] | 202 | 4.30E-09 | 2.17E-07 | 6.66354 |
| LION:0000013 | glycerophosphoserines [GP03] | 42 | 0.00197 | 1.42E-02 | 1.847712 |
| LION:0012085 | golgi apparatus | 12 | 0.02277 | 8.85E-02 | 1.053057 |
| LION:0000093 | headgroup with negative charge | 117 | 2.00E-05 | 5.05E-04 | 3.296709 |
| LION:0000094 | headgroup with neutral charge | 20 | 1 | 1.00E+00 | 0 |
| LION:0000095 | headgroup with positive charge / zwitter-ion | 118 | 0.01805 | 7.60E-02 | 1.119186 |
| LION:0080971 | high bilayer thickness | 17 | 0.33684 | 5.87E-01 | 0.231362 |
| LION:0080979 | high lateral diffusion | 30 | 0.00616 | 3.27E-02 | 1.485452 |
| LION:0001738 | high transition temperature | 19 | 0.55834 | 7.58E-01 | 0.120331 |
| LION:0012084 | lipid droplet | 19 | 1 | 1.00E+00 | 0 |
| LION:0012011 | lipid storage | 19 | 1 | 1.00E+00 | 0 |
| LION:0012009 | lipid-mediated signalling | 177 | 0.61681 | 7.89E-01 | 0.102923 |
| LION:0080969 | low bilayer thickness | 29 | 0.03943 | 1.33E-01 | 0.876148 |
| LION:0080977 | low lateral diffusion | 17 | 0.67946 | 8.20E-01 | 0.086186 |
| LION:0001736 | low transition temperature | 34 | 0.05767 | 1.66E-01 | 0.779892 |
| LION:0000599 | lysoglycerophospholipids | 16 | 0.70173 | 8.21E-01 | 0.085657 |
| LION:0012010 | membrane component | 212 | 1.30E-10 | 1.31E-08 | 7.882729 |
| LION:0012081 | mitochondrion | 63 | 0.00865 | 4.16E-02 | 1.380907 |
| LION:0000034 | monoacylglycerophosphocholines [GP0105] | 9 | 0.26425 | 5.45E-01 | 0.263603 |
| LION:0000042 | monoacylglycerophosphoethanolamines [GP0205] | 3 | 0.67326 | 8.20E-01 | 0.086186 |
| LION:0002969 | monounsaturated fatty acid | 15 | 0.7151 | 8.21E-01 | 0.085657 |
| LION:0012441 | N-acylsphingosines (ceramides) [SP0201] | 71 | 0.7109 | 8.21E-01 | 0.085657 |
| LION:0000464 | negative intrinsic curvature | 187 | 0.50281 | 7.38E-01 | 0.131944 |
| LION:0000465 | neutral intrinsic curvature | 107 | 5.00E-05 | 1.01E-03 | 2.995679 |
| LION:0012082 | plasma membrane | 207 | 0.42766 | 6.86E-01 | 0.163676 |
| LION:0002967 | polyunsaturated fatty acid | 26 | 0.07542 | 1.95E-01 | 0.709965 |
| LION:0000466 | positive intrinsic curvature | 45 | 0.00267 | 1.69E-02 | 1.772113 |
| LION:0002968 | saturated fatty acid | 27 | 0.62613 | 7.90E-01 | 0.102373 |
| LION:0000004 | sphingolipids [SP] | 164 | 0.53086 | 7.48E-01 | 0.126098 |
| LION:0000622 | triacylglycerols [GL0301] | 18 | 1 | 1.00E+00 | 0 |
| LION:0080972 | very high bilayer thickness | 16 | 0.27491 | 5.45E-01 | 0.263603 |
| LION:0080980 | very high lateral diffusion | 21 | 0.00013 | 2.19E-03 | 2.659556 |
| LION:0001739 | very high transition temperature | 19 | 0.00952 | 4.37E-02 | 1.359519 |
| LION:0080968 | very low bilayer thickness | 15 | 0.00047 | 4.75E-03 | 2.323306 |
| LION:0080976 | very low lateral diffusion | 19 | 0.14197 | 3.27E-01 | 0.485452 |
| LION:0001735 | very low transition temperature | 18 | 0.01442 | 6.33E-02 | 1.198596 |
